# Regulatory T cells and their role in allergic disease

**DOI:** 10.1111/all.16326

**Published:** 2024-09-27

**Authors:** Melanie L. Conrad, Gabriela Barrientos, Xuejun Cai, Saikat Mukherjee, Mrinmoy Das, Emmanuel Stephen‐Victor, Hani Harb

**Affiliations:** ^1^ Institute of Microbiology, Infectious Diseases and Immunology, Charité Universitätsmedizin Berlin, Corporate Member of Freie Universität Berlin Humboldt‐Universität zu Berlin Berlin Germany; ^2^ Institute of Medical Psychology, Charité Universitätsmedizin Berlin, Corporate Member of Freie Universität Berlin Humboldt‐Universität zu Berlin Berlin Germany; ^3^ Neonatology and Pediatric Intensive Care, Faculty of Medicine University of Augsburg Augsburg Germany; ^4^ Laboratory of Experimental Medicine Hospital Alemán Buenos Aires Argentina; ^5^ National Scientific and Technical Research Council (CONICET) Buenos Aires Argentina; ^6^ Institute of Medical Microbiology and Virology, Medical Faculty, TU Dresden University Hospital Dresden Dresden Germany; ^7^ Division of Immunology Boston Children's Hospital Boston Massachusetts USA; ^8^ Department of Pediatrics Harvard Medical School Boston Massachusetts USA; ^9^ Department of Pathology, Division of Microbiology and Immunology, Huntsman Cancer Institute University of Utah School of Medicine Salt Lake City Utah USA

**Keywords:** allergy, asthma, atopic dermatitis, food allergy, T regulatory cell

## Abstract

The incidence of allergic diseases has been rising over the past decades, and this troubling trend coincides with environmental changes such as shifts in diet and increased antibiotic use, both of which can impact our immune system. Allergic reactions occur when the immune system overreacts to normally harmless substances, and it is known that regulatory T cells (Tregs) play a major role in immune system suppression and the generation of tolerance. However, new research suggests that Tregs can malfunction in environments that promote allergies. This review delves into Treg function, and how environmental factors can influence their ability to maintain immune homeostasis. Specifically, we explore the origins of Treg cells, as well as the mechanisms used for suppression of inflammation and tissue healing, with a concentration on food allergies, atopic dermatitis and asthma. Understanding Treg function in the context of a changing environment is crucial for developing new strategies to prevent and treat allergies.

**Abbreviations**
AD: Atopic dermatitis
Ahr: Aryl hydrocarbon receptor
APC: Antigen presenting cells
Areg: Amphiregulin
CagA: cytotoxin‐associated gene A
CRTH2: Chemoattractant receptor‐homologous molecule expressed on Th2 cells
CTLA‐4: Cytotoxic T lymphocyte antigen 4
DC: Dendritic cell
DEREG: DEpletion of REGulatory T cells
FA: Food allergy
FOXP3: Forkhead box P3
GATA3: GATA‐binding protein 3
GGT: γ‐glutamyl transferase (GGT)
HDM: House dust mite
IgE: Immunoglobulin E
IL: Interleukin
ILC: Innate lymphoid cell
IPEX: Immunodysregulation, polyendocrinopathy, enteropathy, X‐linked
iTreg: Induced T regulatory cell
LAG‐3: Lymphocyte activation gene 3
MI: Myocardial infarction
mLN: Mesenteric lymph nodes
nTreg: Natural T regulatory cell
PAH: Polycyclic aromatic hydrocarbons
PM: Particulate matter
PSA: Polysaccharide A
RALDH2: Retinoic acid catalysing enzyme
ROR𝛾t: RAR‐related orphan receptor gamma t
RyR2: Ryanodine receptor
SAG: Staphylococcal enterotoxin B
SCFA: Short chain fatty acid
SNP: Single nucleotide polymorphism
Tbet: T‐box transcription factor
TCR: T‐cell receptor
Teff: Effector T cell
Tfh: T follicular helper
Tfr: Follicular T regulatory
TGF‐β: Transforming growth factor beta 1
Th: T helper
Th2: Type 2 T helper cell
Tr1: T regulatory 1
Treg: Regulatory T cell
VacA: vacuolating cytotoxin A

## 1 INTRODUCTION

The escalating incidence of allergic disorders has become a pressing health issue in affluent and rapidly developing societies.^1^, ^2^ Furthermore, the Industrial Revolution's transformative social and environmental changes, significantly impacting human behaviour, lifestyle, diet and infectious exposures over the past 150 years, have contributed to the rise and severity of allergic diseases.^2^, ^3^, ^4^, ^5^ In the United States, food allergy prevalence is 8% in children and 5% in adults, while asthma affects 8.6% of children and 7.4% of adults.^6^ Additionally, in the context of early childhood, there is evidence of the allergic march, which begins with atopic dermatitis, then progresses to food allergies, allergic rhinitis and asthma.^7^ This amplified burden of allergic diseases has led to considerable morbidity and substantial financial burden for individuals and healthcare systems.^8^ While therapeutic advancements have targeted inflammatory processes and provided symptomatic relief, these therapies are, for the most part, non‐curative.

The dramatic surge in allergic disease prevalence in recent decades strongly suggests an influence of environmental factors that interact with genetically predisposed individuals to promote disease development.^9^ Recent studies emphasize a dynamic interplay of environmental factors including: diet,^10^ pollutants,^11^ nonpathogenic bacterial exposure^12^, ^13^ and antibiotic use^14^, ^15^, ^16^ on immune system development and function.^17^, ^18^ These findings also support the central role of commensal bacteria in regulating allergic diseases, through dynamic interaction with the host's genetic background and environmental inputs that either foster or disrupt tolerance mechanisms.^12^, ^19^, ^20^, ^21^, ^22^, ^23^

Considering immune tolerance, it is well known that regulatory T (Treg) cells play a crucial role in promoting tolerance to allergens and preventing allergic disease.^24^, ^25^ This review will delve into recent advancements highlighting the ‘dual potential’ of Tregs in allergic disease, namely their ability to either promote tolerance in a healthy environment or contribute to disease exacerbation when exposed to a pro‐allergic inflammatory milieu.

## 2 TREGS—ORIGIN AND MECHANISM OF ACTION

**FIGURE 1:**
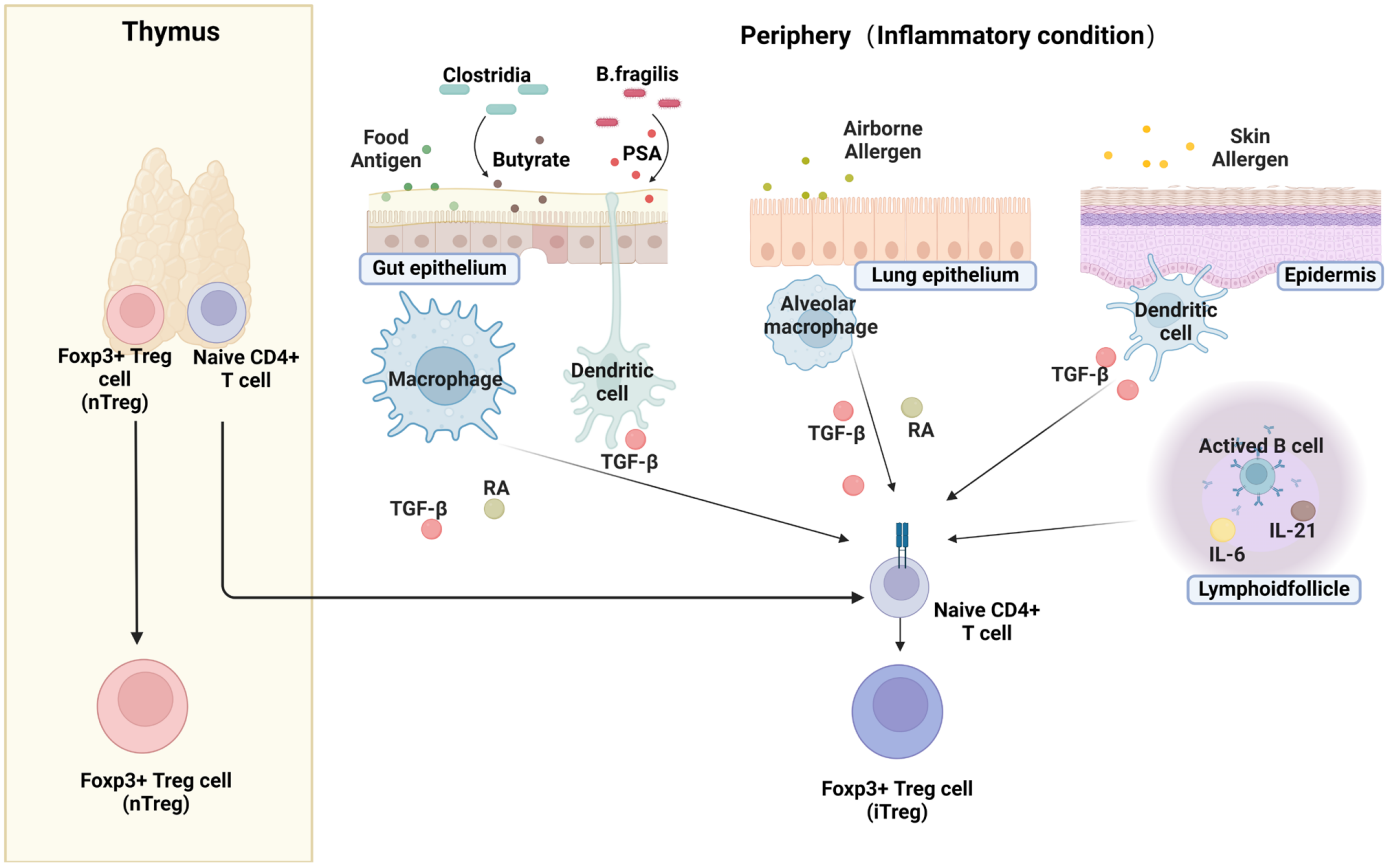
Origins of Treg cells. Treg cells originate either from the thymus (called natural Tregs (nTreg)) or develop in the periphery from naïve CD4+ T cells into induced Tregs (iTreg). nTregs mediate self‐antigen tolerance, whereas iTregs develop in peripheral tissues and play an important role in maintaining tolerance at mucosal surfaces. iTregs differentiate in the gut via: Stimulation by TGF‐β and retinoic acid, exposure food allergens and contact with bacterially produced SCFAs or bacterial components such as *Bacteroides*‐derived polysaccharide A (PSA). In the lungs, iTregs can be induced by airborne allergens as well as TGF‐β and retinoic acid secreted by alveolar macrophages. Finally, in the skin, iTregs can be activated by contact with skin allergens and through dendritic cell (DC) TGF‐β production.

Treg cells are divided into two major categories: the natural (nTregs) (also called thymic Tregs) and the induced (iTreg) (also called peripheral Tregs),^26^, ^27^, ^28^ shown in Figure 1. nTregs are thymus‐derived and mainly mediate self‐antigen tolerance, whereas iTregs (derived from naïve CD4 T cells in the peripheral blood) become tissue resident cells that play a crucial role in homeostasis and the regulation immune responses within particular organs.^26^, ^27^ For example, iTreg cells in the gut and the lung can be induced via microbially derived metabolites,^29^ TGF‐β and retinoic acid,^30^, ^31^ shown in Figure 1. Additionally, iTreg cells can also be induced in the gut, to protect from food allergy.^32^, ^33^ Though both nTregs and iTregs use similar immune suppressive mechanisms, major differences are observed in their T‐cell receptor (TCR) repertoire. iTregs recognize a much wider array of antigens and thus have better ability to engage pathogens, allergens and other factors encountered in the peripheral tissues.^34^

**FIGURE 2:**
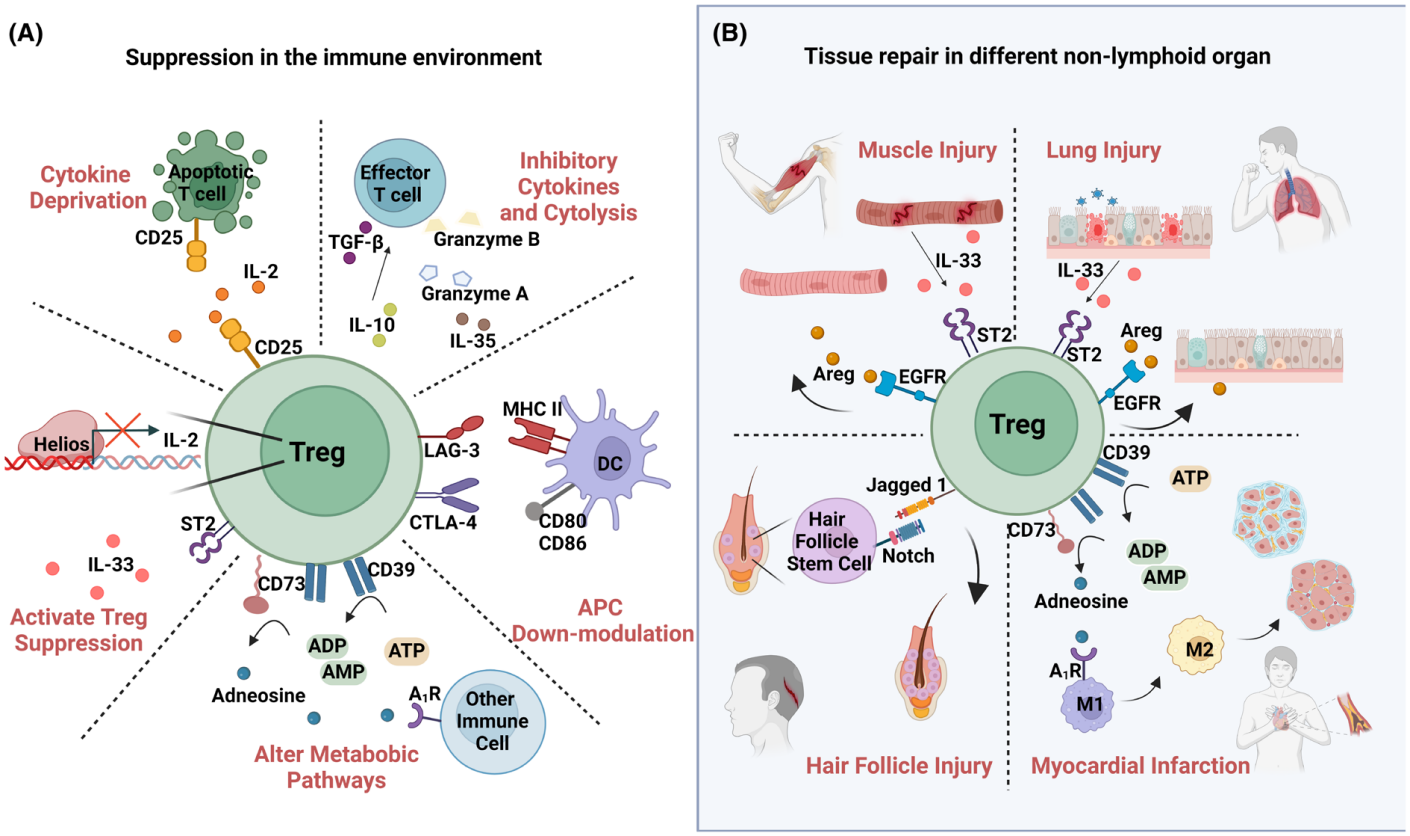
Mechanisms of action in Treg cells. (A) Treg cells suppress immune responses through multiple mechanisms. Tregs can secrete cytokines such as IL‐10, TGF‐β and IL‐35, as well as cytolytic factors such as granzyme A and B to mediate suppressive function. Regarding cell–cell contact, binding of CTLA‐4 and LAG‐3 on Tregs to B7 and MHCII molecules on APCs results in inhibition of APC activation. Tregs convert ATP to adenosine via CD39 and CD73, reducing Teff cell proliferation and pro‐inflammatory cytokine production. IL‐33 binds to ST2 on Tregs, enhancing their regulatory and suppressive functions in the lungs and colon. The transcription factor Helios inhibits IL‐2 expression in Tregs. Additionally, IL2/CD25 on Tregs suppress APCs by depriving them of essential cytokines. (B) Tissue repair mechanisms in Treg cells. Treg cells aid tissue repair by producing Areg in response to IL‐33 from muscle or lung injury, promoting repair and cell proliferation. In myocardial infarction, Tregs convert ATP to adenosine, transforming M1 macrophages to an anti‐inflammatory M2 phenotype which produces IL‐10. Tregs also promote hair growth in skin via the Notch pathway.

Tregs play an important suppressive role in the immune response, by acting at sites of inflammation to control T effector (Teff) cell function through humoral factors or via direct cell–cell interactions. In the case of humoral suppression, IL‐10 secretion inhibits Teff activation, while TGF‐β and IL‐35 promote Treg differentiation and enhance function. Additionally, secretion of cytolytic factors such as granzyme A and B induce apoptosis in Teff cells^35^, ^36^, ^37^, ^38^; however, it was also shown that granzymes can be self‐damaging to the cells that secrete them, as Tregs as cells producing granzyme B were shown to be more apoptotic.^39^ Considering cell–cell interactions, IL2/CD25, as well as several immune checkpoint receptors including: cytotoxic T lymphocyte antigen 4 (CTLA‐4), lymphocyte activation gene 3 (LAG‐3), CD73, CD39 and ST2, participate in the Treg mechanism of action, illustrated in Figure 2A. IL2/CD25 interaction promotes the suppression of antigen presenting cells (APCs) by depriving them of their trophic cytokines IL‐4, IL‐10 and IFN𝛾.^40^ Additionally, the transcription factor Helios (a repressor of Treg IL‐2 expression) has been identified as a pivotal component for the suppressive capacity and stabilization of Treg cells during inflammatory processes.^41^ Considering checkpoint inhibitors, binding of CTLA‐4 on Tregs to the B7 ligands (CD80 and CD86) on Teff cells inhibits their activation and proliferation,^42^ whereas binding of LAG‐3 to MHCII molecules causes cell exhaustion.^43^ Both CTLA‐4 and LAG‐3 expression on Tregs also down‐modulate APCs in a similar fashion.^42^, ^44^ Tregs also exert their function through membrane bound enzymes such as CD39 and CD73 which act together to convert extracellular ATP to immunosuppressive adenosine. The binding of adenosine to receptors on Teff cells reduces T‐cell proliferation and dampens the production of pro‐inflammatory cytokines. Notably, generation of adenosine by these enzymes can also lead to loss of Treg suppressive ability.^45^ Finally, the IL‐33 receptor (ST2) acts as an activator of Treg cells. Upon release by epithelial cells, IL‐33 binds to ST2, magnifying the regulatory and suppressive capacity of Treg cells both in the lungs and the colon.^22^, ^46^

The suppressive function of Treg cells is not the only component of their regulatory profile. These cells also participate in tissue repair mechanisms, which are crucial to orchestrate the healing response after severe inflammatory responses. For instance, injured muscles produce IL‐33, which subsequently stimulate Tregs to produce amphiregulin (Areg), a molecule which stimulates tissue repair and proliferation of satellite cells through the epidermal growth factor receptor pathway.^47^ It is known that Areg, expressed on cytotoxic CD4+ and CD8+ T cells, plays a protective role in the pathogenesis of fibrotic disorders^48^ and bacterial infections.^49^ In allergic disease, Areg expression on CCR10+ ILC2 cells also plays an important role protecting against allergic asthma.^50^ Additionally, in the lungs, Treg cells produce Areg in response to lung injury following viral lung infection.^51^ Nevertheless, Areg is not always a protector. There have been reports that Areg induction in the skin of mice with mutated keratin (and defective barrier function) promoted itching through thymic stromal lymphopoietin (TSLP) production by keratinocytes.^52^ In other types of tissue damage, such as in myocardial infarction (MI), Treg cells play an important role in repair processes though CD39‐mediated adenosine formation, which stimulates switching of M1 macrophages towards an anti‐inflammatory M2 phenotype.^53^, ^54^, ^55^ The recruitment of Treg cells into the heart tissues is CCR5 dependent, as shown in knocking down CCR5 in mice, which led to impaired healing due to impaired Treg infiltration.^56^, ^57^ Tissue repair processes of Treg cells are shown in Figure 2B.

## 3 TREG PLASTICITY AND EPIGENETICS

Tregs display a great deal of plasticity, using their cytokine and chemokine profiles to control inflammatory processes via the suppression of various immune cell subtypes. For instance, in Peyer's patches in the murine gut, expression of IL‐6 and IL‐21 can stimulate the transformation of Tregs into follicular Th (Tfh)‐like cells called follicular Tregs (Tfr).^58^, ^59^, ^60^ These regulatory Tfr migrate to the germinal centre, promoting germinal centre formation and inhibiting Tfh‐mediated B‐cell activation and antibody production,^61^, ^62^, ^63^ shown in Table 1. Moreover, in addition to FOXP3, Tregs can also co‐express T‐box transcription factors (T‐bet), forming Th1‐like Tregs. These cells can help suppress excessive Type 2 response associated with allergy through the secretion of IFN𝛾 and their maintenance of suppressive function^64^, ^65^, ^66^, ^67^ (Table 1).

**TABLE 1:** Treg subtypes.

Name	Cellular markers	Role in the immune response
Follicular Tregs (Tfr)	FoxP3+ CD25+ CXCR5+ BCL6+ PD1+ CTLA4+ ICOS+	Migration to germinal centre, inhibition of Tfh‐mediated B‐cell activation and antibody production
Th1‐like Tregs	FoxP3+ CD25+ CD127low T‐bet+ CCR5+ CXCR3+	Increased IFN*γ* secretion that may help suppress excessive Type 2 responses
Th17‐like Tregs	FoxP3+ CD25low CD127low ROR*γ*t+ CCR6+	Enhance Th17 responses in the lung that aggravates allergic asthma. In contrast in the gut, they suppress food allergy
Th2‐like Tregs	FoxP3+ CD25+ CD127low GATA3+ CCR4+	Expression of IL‐4, IL‐5 and IL‐13 exacerbates allergic asthma and food allergy
exTregs	FoxP3low CD25− CD127+	Transition from FoxP3 expressing suppressive cell to inflammatory effector T cell. Worsens allergic inflammation

Although Treg plasticity underpins a well‐regulated immune response, exposure to chronic inflammation can also shift these responses from a suppressive to an inflammatory phenotype. For instance, gut resident Tregs that co‐express the RAR‐related orphan receptor gamma t receptor (ROR𝛾t), known as Th17‐like Tregs, suppress food allergic responses, but in contrast promote allergic asthma in the lungs via IL‐17 production.^19^, ^68^, ^69^, ^70^, ^71^ Tregs co‐expressing FoxP3, GATA‐binding protein 3 (GATA3) and a receptor known as ‘chemoattractant receptor‐homologous molecule expressed on Th2 cells’ (CRTH2) participate in ILC2 recruitment to the lungs.^72^ This influx of ILC2 cells combined with the production of IL‐4, IL‐5 and IL‐13 from these Th2‐like Tregs worsens allergic asthma and food allergy.^72^, ^73^, ^74^, ^75^, ^76^ Finally, continued exposure to a chronic inflammatory milieu can lead to loss of FoxP3 expression, in a process that is not yet completely understood. This results in a cell type called exTregs, consisting of former Tregs that have lost their suppressive capabilities and effectively transitioned into conventional T effector cells that participate in pathogenic immune reactions and exacerbate allergic inflammation.^77^

Epigenetic mechanisms, such as DNA methylation and histone modification, play a crucial role in regulating Treg plasticity at key genomic loci. For instance, nTregs can be distinguished from iTregs by a significant DNA hypomethylation at the Foxp3 promotor and Foxp3‐associated enhancer regions such as the Treg cell‐specific demethylated region (TSDR—also known as conserved noncoding DNA sequence 2 (CNS2)).^78^, ^79^ Furthermore, maintenance of DNA methylation by DNA methyltransferase DNMT1 and Ten‐Eleven Translocation (TET) enzymes is required to control the stability of Foxp3 in nTregs in thymus, as deletion of this gene results in diminished numbers and suppressive function of Treg cells.^80^ Thus, nTreg cells require both a canonical hypomethylation pattern and maintenance methylation to stabilize their lineage identity and function. In addition to methylation, the FoxP3 locus is also acetylated during T‐cell development. Histone acetyltransferases (HATs) interact with the FoxP3 locus during this time to promote sustained FoxP3 expression in Treg cells.^81^, ^82^

A chronic inflammatory milieu is also a strong environmental driver of epigenetic changes, and modifications to the FoxP3 locus directly influence T reg plasticity. For example, if TET or HAT enzyme activity is lost, due to an infection or a metabolic change, Tregs lose Foxp3 expression and gain a Th17‐like phenotype.^82^ In addition to this, a sustained level of IL‐6 can lead to loss of Foxp3 by promoting DNMT1‐mediated DNA methylation and histone deacetylase (HDAC) activity.^83^ The substantial epigenetic regulation involved in Treg plasticity opens up promising avenues for future therapeutic interventions, where targeting these epigenetic mechanisms could potentially modulate Treg function in allergic diseases. Descriptions of epigenetic modifiers of Treg plasticity are shown in Table 2.

**TABLE 2:** The mechanism and the role of epigenetic modifiers in Treg cells.

Epigenetic modifier	Mechanism	Role in Treg cells
TET enzymes	DNA demethylases	Induce and maintain Foxp3 expression
HATs	Histone acetylation enzymes	Induce and maintain Foxp3 expression
Satb1	Genome organizer	Stabilizes the Treg cell‐defining gene regulatory network
CoREST	Epigenetic repressor complex	Disrupts Foxp3‐driven repression of Th1 cytokines
UHRF1	DNA methytransferase adapter protein	Controls stability of Foxp3 in nTregs in the thymus
DNMT1	Maintenance DNA methyltransferase	Stabilizes lineage identity and function of Tregs
EZH2	Histone methytransferase	Binds to Foxp3 containing domains and deposits a repressive chromatin modification

## 4 TREG CELLS AT BARRIER SITES

The epithelial barrier is an important component to consider in the pathogenesis of allergic disease.^84^, ^85^ The epithelium of the skin, gastrointestinal tract and lungs engage in intricate interactions with the environment, microbiota and immune system, and disruption at barrier sites is associated with altered immune homeostasis^86^, ^87^, ^88^, ^89^ and the development of allergic diseases such as asthma,^90^ atopic dermatitis,^91^ allergic rhinitis,^92^ chronic rhinosinusitis^93^ and eosinophilic esophagitis.^94^ Interestingly, disrupted barrier function in specific organs can also influence the development of allergic diseases in different sites. The gut–lung axis provides an excellent example of such barrier dysfunction wherein early‐life gut microbial dysbiosis has been linked to increased asthma severity.^21^ Tregs play a pivotal role in maintaining epithelial barrier homeostasis through several different pathways.^95^ In the intestine, Tregs can promote intestinal barrier integrity via the secretion of IL‐10,^96^ suppression of neutrophil infiltration^97^ and inhibition of type 2 immune responses in food allergies.^98^ Finally, in the lung, Tregs promote epithelial proliferation in a CD103‐depdendent manner.^99^

## 5 TREG CELLS IN SKIN DISEASE

Atopic dermatitis (AD) or eczema is a prevalent inflammatory skin disease, characterized by abnormalities in the skin barrier, cellular immune deviations and increased sensitization to environmental allergens, that can affect both children and adults.^100^ AD lesions are characterized by the infiltration of activated Th2 cells and eosinophils, expansion of ILC2 cells, production of IL‐4 and IL‐13 as well as elevated levels of total and allergen‐specific IgE, all of which are directly associated with disease severity.^101^, ^102^, ^103^, ^104^ Regarding Tregs, these cells are highly enriched in the skin of humans and mice and are important for the control of allergic inflammation and repair.

**FIGURE 3:**
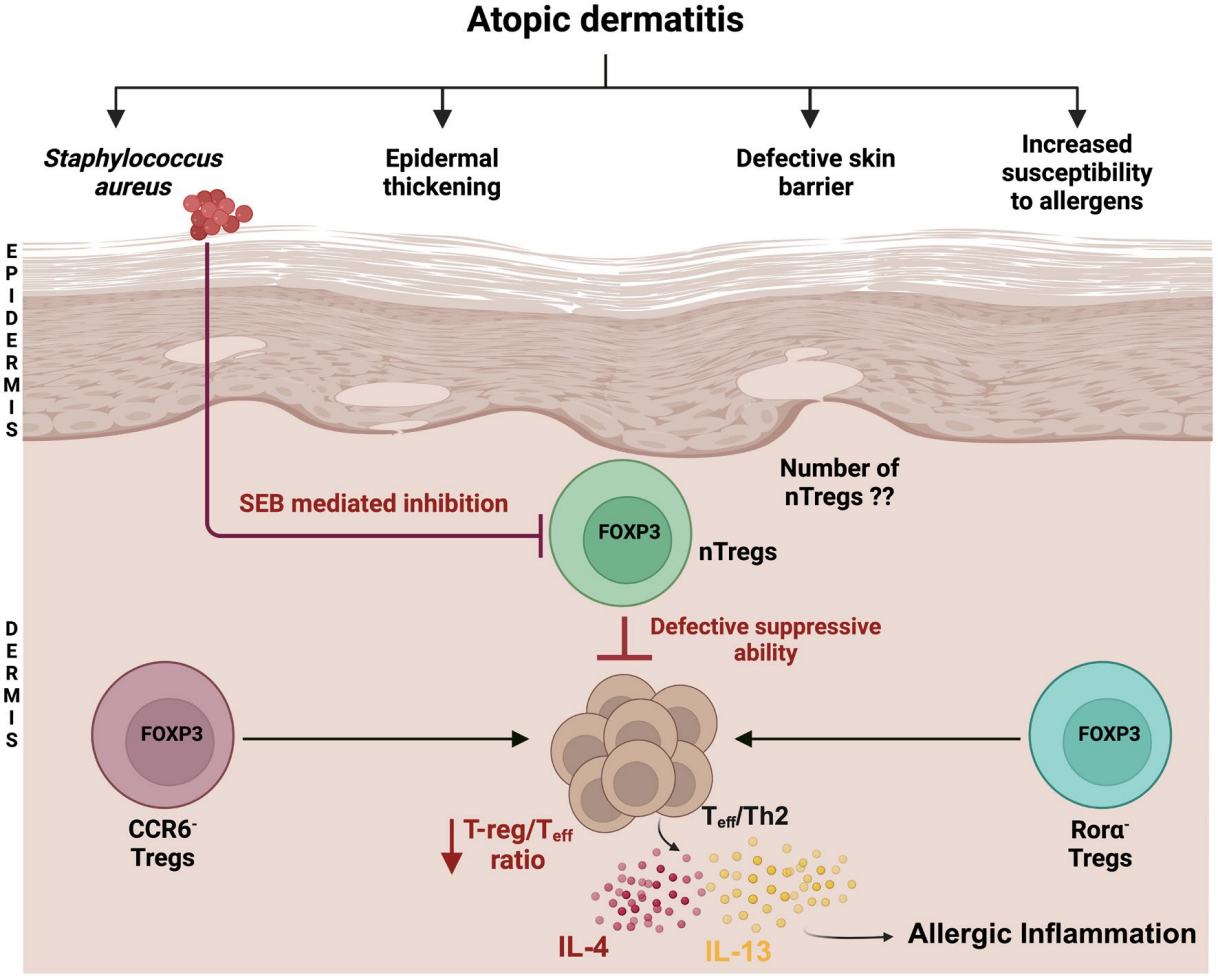
Dysregulation of Tregs in atopic dermatitis. AD is characterized by epidermal thickening, skin barrier defects and increased susceptibility to allergen. Though the number of Tregs residing in the skin during AD patients is not clear, it is known that the suppressive ability of Tregs is defective due to a lower ratio of Tregs to Teff cells (Treg/Teff). Subpopulations of Rorα‐ and CCR6‐Tregs are also involved in AD and through expression of IL‐4 and IL‐13 can promote Th2 immune responses in the skin. Additionally, colonization of the skin by *S. aureus* is highly prevalent in AD patients, inhibiting Treg activity through the production of superantigens such as SEB.

The most striking evidence for Tregs in protection against AD is observed in the IPEX disease, in humans (associated with FOXP3 gene mutations)^105^ and in scurfy mice (which lack Treg cells), which is characterized by severe allergic skin inflammation, mimicking the skin lesions observed in AD. In addition to this, CARMIL2 deficiency (capping protein regulator and myosin 1 linker 2) is an autosomal recessive inborn error of immunity (IEI) that causes dysfunction in T‐cell activation and decrease in the Treg population, which is also associated with AD.^106^ Tregs have been shown to attenuate skin inflammation in several mouse models of AD, for instance, Nidhi et al. showed that RORα‐expressing skin Tregs were important in suppressing type 2 cytokines in an ILC2 model of AD.^107^ Furthermore, sonic Hedgehog (Shh) signalling in skin reduced AD pathology by increasing Treg‐mediated immune suppression.^108^ Moreover, defective Treg function underlies the exaggerated contact hypersensitivity exhibited by *Dock8* ^*−/−*^ mice in response to the hapten oxazolone, and increases their susceptibility to allergic skin inflammation elicited by skin sensitization with antigen or by cutaneous exposure to *Staphylococcus aureus*,^109^, ^110^, ^111^ shown in Figure 3.

Despite the well‐established presence of Tregs in AD skin, the recruitment of these cells and their potential dysfunction in AD remain perplexing. It is known that an elevation of Treg cells significantly correlates with AD severity, however while some studies show Treg accumulation in skin lesions, but no increase in the number of circulating cells^112^ others showed increased Treg presence in both the blood and skin of AD patients^113^, ^114^, ^115^, ^116^, ^117^ or decreased frequency of circulating T regs.^118^ Furthermore, a recent meta‐analysis study showed increased Th17/Th22 cells and decreased Treg cells in blood of AD patients.^119^ These discrepancies could be due to the plasticity of Treg cells and the markers employed for cell identification. Importantly, FOXP3 expression does not occur exclusively in Tregs, it can also be transiently induced in activated skin Teff cells.^120^, ^121^, ^122^

Treg expresses a variety of skin‐homing addressins including CCR4, CCR5, CCR6 and cutaneous lymphocyte‐associated antigen (CLA),^105^, ^123^, ^124^, ^125^ and several reports have identified increased expression of these molecules on skin Treg cells in AD.^115^, ^124^, ^126^ Additionally, there is a subpopulation of skin Treg cells that lack expression of CCR6 and promote the Th2 immune response.^105^, ^125^, ^127^, ^128^ Despite the augmented number of Treg cells in the skin of AD patients, a decreased Treg/Teff ratio still results in higher levels of IL‐4 and IL‐13,^129^ shown in Figure 3. In addition to quality and quantity impairments, Treg cells also exhibit high plasticity. Treg cells able to differentiate into Th1, Th2 or Th17 under the influence of specific cytokines and by epigenetic reprogramming,^105^, ^130^, ^131^ thus repressing their suppressive functions and contributing to AD pathogenesis.

Regarding bacterial colonization, increased Th2 cytokines and decreased antimicrobial peptide synthesis facilitates *S. aureus* skin colonization in AD patients compared to healthy controls, and the load of *S. aureus* correlates with disease severity.^132^ *S. aureus* strains produce superantigens such as staphylococcal enterotoxin B (SEB), and several reports have shown that these superantigens inhibit Treg suppressive function and promote Th2 like functions.^133^ This SEB‐mediated Treg inhibition was shown to be modulated through the glucocorticoid‐induced tumour necrosis factor receptor family‐related protein (GITR/GITRL) pathway and upregulation of the GITR ligand on monocytes.^134^ Thus, Treg impairment may be more prominent in AD patients with *S. aureus* colonization. In contrast, the presence of the commensal bacterium *Lactobacillus rhamnosus* activates Treg function and suppresses Th1, Th17 and thymic stromal lymphopoietin‐mediated responses in AD patients.^126^ Several treatments have been developed for AD patients that either generate or modulate Treg cell function. Low‐dose IL‐2 treatment, which has the potential to increase Treg number and function, showed an improvement of clinical symptoms and signs in eczema in AD patients.^135^ Treatment with allergen immunotherapy (AIT) and vitamin D supplements, both of which have the potential promote Treg cell development, also showed a significant reduction in the severity of AD.^136^

In summary, the role of Tregs in the pathogenesis of AD is complex and remains an area of investigation. Comprehensive phenotypic and mechanistic studies investigating Tregs during AD skin flares are required to better illuminate their role in this disease, and additionally, to develop therapeutics harnessing Tregs for AD treatment. From a clinical standpoint, the mechanisms by which Treg cells contribute to AD initiation are an important issue to be addressed.

## 6 TREG CELLS IN FOOD ALLERGY

The gastrointestinal tract is continuously exposed to foreign antigenic material derived from the diet, and more than 100 g of food protein from plant and animal sources is consumed every day. Despite this enormous load of foreign antigens, in most individuals the immune system does not mount an adverse immune reaction to food proteins, primarily due to the induction of oral tolerance. Oral tolerance is the default physiological mechanism that is actively induced in response to orally ingested foreign antigens, and it is the underlying basis through which large amounts of food antigens can be consumed every day. However, breakdown of oral tolerance triggers a pathogenic type 2 immune response characterized by the generation of high‐affinity IgE responses to food antigens. In individuals sensitized to food proteins, subsequent exposure to the sensitized food results in mast cell and basophil activation and in rare cases such a response results in anaphylaxis, an acute life‐threatening systemic allergic response to food.^137^, ^138^, ^139^, ^140^ Consequently, the diagnosis of food allergy (FA) is carried out through an allergen skin prick test and the detection of food allergen‐specific IgE levels in the serum.^141^, ^142^, ^143^ Several mechanisms have been described in regulating oral tolerance including the induction of T‐cell anergy, clonal deletion of effector T cells, induction of IgG and IgA responses and the generation of iTreg cells.^19^, ^144^, ^145^

Immune exposure to large doses of food antigens has been shown to induce T‐cell anergy and clonal deletion of antigen reactive T cells via apoptosis.^146^ Conversely, low and sustained doses of food antigens induce antigen‐specific Treg cells.^146^ A recent study explored the fate of the CD4 T‐cell response to gliadin,^78^ a major wheat protein that harbours IgE epitopes and is known to cause a spectrum of allergies such as atopic eczema and food allergy.^147^ Mice fed with gliadin induced a small proportion of T follicular helper (Tfh) like cells that expressed CXCR5 and promoted a weak anti‐gliadin IgG1 response.^148^ Gliadin exposure also induced a significant non‐canonical anergic T helper cell subset that expressed the folate receptor and CD73. These anergic T helper cells lacked inflammatory functions and were incapable of inducing gut pathology, eventually differentiating to Treg cells (under the influence of IL2).^148^ Allergen exposure in general has also been shown to induce IL2 production in effector Th2 cells, subsequently promoting the maintenance and survival of Treg cell responses which in turn suppresses pathogenic allergen‐specific Th2 cells.^149^ Therefore, low‐dose IL2 has been proposed as a key therapeutic strategy in controlling FA.^150^, ^151^ Finally, the role of iTregs in regulating tolerance to food antigens has also been described using DEREG mice (DEpletion of REGulatory T cells), which express the diphtheria toxin receptor driven by the FOXP3 promoter. Depletion of Treg cells with diphtheria toxin after the induction of oral tolerance to food antigens resulted in mice developing antigen‐specific IgE responses and food allergy,^152^ corroborating a necessary role for iTregs cells in controlling tolerance to food antigens.^153^

**FIGURE 4:**
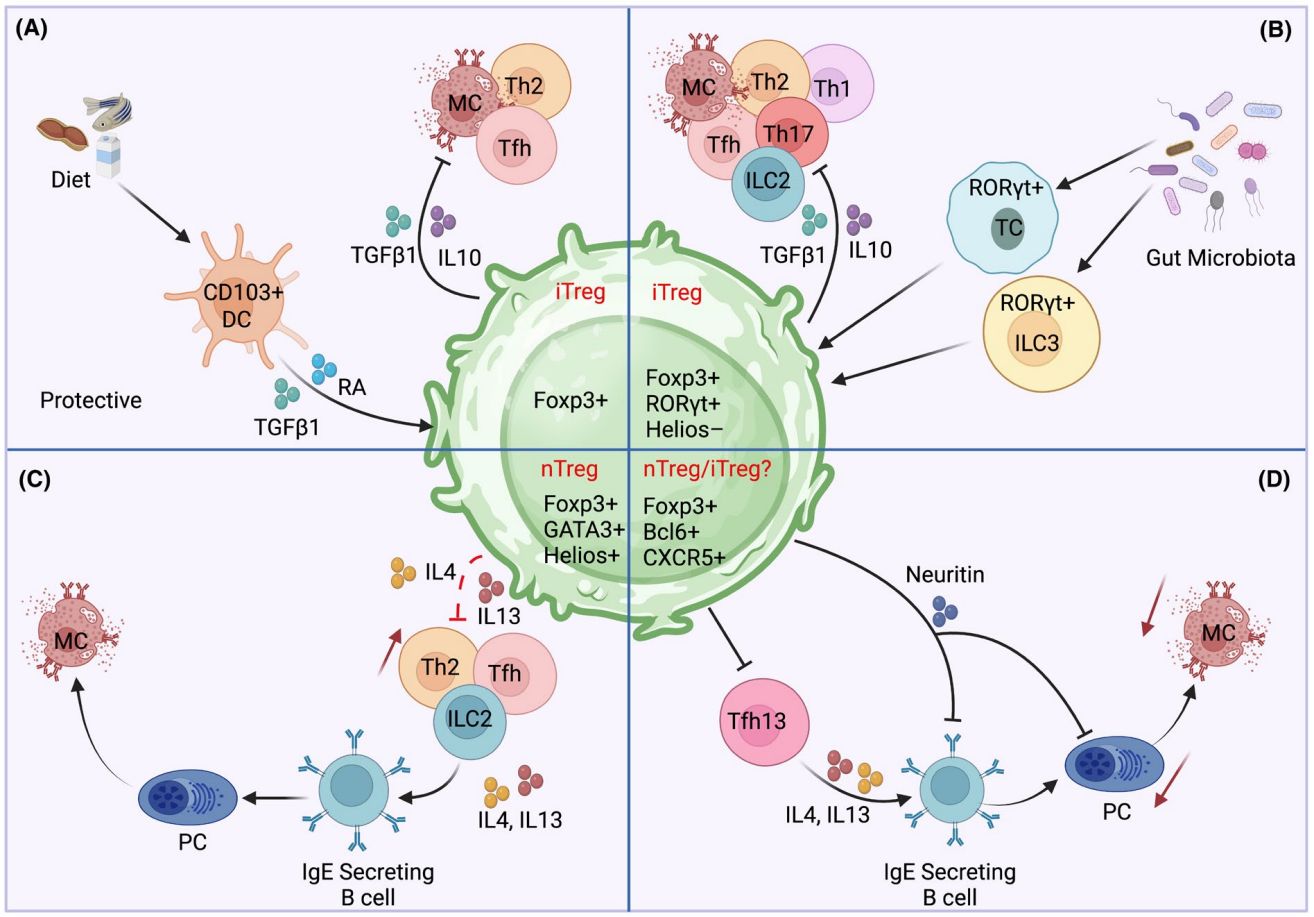
Treg cell subsets in food allergy (FA). (A) Classical antigen presenting cells (APCs) such as CD103+ dendritic cells (DCs) capture food antigens and induce the differentiation of antigen‐specific Treg cells under the influence of TGF‐β1 and retinoic acid. iTreg cells secrete IL‐10 and TGF‐β1 and suppress Th2 responses to food antigens. (B) ROR𝛾t + APCs, including type 3 innate lymphoid cells (ILC3s) and Thetis cells (TC), sample commensal antigens in the gut lumen and induce ROR𝛾t+ iTreg cells. Commensal‐induced ROR𝛾t + Treg cells suppresses allergen‐specific Th2 responses and mast cell activation through a TGF‐β1 dependent mechanism. (C) Impairment in the microbiota‐food‐antigen‐Treg axis disrupts the tolerogenic balance in the gut and promotes the expansion of Th2‐cell‐like Tregs which express GATA3 and secrete IL‐4 and IL‐13. These Th2‐reprogrammed Treg cells fail to curtail allergen‐specific immune responses leading to a dysregulated Th2 and Tfh responses that promote IgE antibody production and mast cell activation to food. (D) Tfr cells limit the pathogenic activity of Tfh cells and secrete neuritin which repress IgE class switching and plasma cell formation in B cells.

Tregs specific for food antigens are induced in the gut‐draining mesenteric lymph nodes (mLN).^154^ Intestinal phagocytes such as macrophages and DCs extend dendrites through enterocytes to sample luminal antigen. Non‐migratory CX3CR1^+^ macrophages capture soluble antigens from the lumen and transfer them to migratory CD103+ DCs via connexin 43 gap junctions.^155^ The small intestine contains different DC subsets, with CD103+ DCs residing in the lamina propria (LP), and exhibiting a mature yet tolerogenic phenotype through the expression of the immunoregulatory cytokines IL‐10, IL‐27, TGF‐β1 and the M2 catalysing enzyme RALDH2. CD103+ DCs migrate to mLN in a CCR7 dependent manner to induce antigen‐specific Treg cells,^154^ shown in Figure 4A. Food‐derived retinoic acid also orchestrates the transmigration of cDC2s from the LP to intraepithelial sites, programming a tolerogenic milieu under the influence of environmental cues and mucin.^156^ Indeed, DCs differentiated in the presence of retinoic acid efficiently suppress allergen‐specific Th2 responses in vitro and in vivo.^157^, ^158^ In addition to regulating the phenotype and functionality of DCs, retinoic acid also plays a critical role in imprinting the gut homing markers CCR9 and integrin α4β7 on Treg cells, which guides them back to the intestine where they proliferate and promote oral tolerance.^159^ Perturbation of this trafficking axis is associated with failure to induce oral tolerance to food antigens, as mice deficient in integrin beta 7 or CCR7 are associated with loss of tolerance to orally administered antigens.^152^, ^154^ Furthermore, eosinophils migrating along the crypt‐villus axis need retinoic acid for their maintenance.^160^ In addition to eosinophils, ILC2 require retinoic acid for a better adaptation to the small intestine environment.^161^

In addition to antigens derived from the diet, the intestinal immune system is constantly interacting with the antigens and immunogenic components derived from the commensal microbiota residing in the gut mucosa. Gut microbial composition is highly dynamic, specifically during the early life where nutritional demands dictate the succession of microbial communities.^162^ Interaction with the gut microbiota in early life influences immune system development, and Treg cell imprinting during this crucial time period is hypothesized to play a role in the suppression of allergic reactions to food antigens.^19^ *Bifidobacteria* and *Lactobacillus* species predominantly colonize the neonatal gut and secrete diverse neurotransmitters that induce Tregs early in life, licensing long‐term tolerance to food antigens later in life.^163^ Then, the introduction of solid foods during the weaning period promotes the expansion of *Clostridia* and Bacteroidetes clades which subsequently induce a highly specialized subset of Treg cells expressing ROR𝛾t.^74^, ^75^

ROR𝛾t Tregs are induced early in life under the direct influence of the gut microbiota and stably persist to adulthood, promoting tolerance to food and commensal antigens by actively suppressing pathogenic Th1, Th2 and Th17 responses,^164^, ^165^ shown in Figure 4B. Interestingly, ROR𝛾t Treg differentiation was found to be governed by MHCII^+^ROR𝛾t^+^ APCs that were distinct from DCs.^166^, ^167^, ^168^ These ROR𝛾t^+^ APCs were characterized as ILC3s, and a newly described cell type named as Thetis cell. Both ILC3s and Thetis cells induced ROR𝛾t Tregs through TGF‐β1 signalling pathways involving αVβ integrin dependent mechanisms, with Thetis cells playing a dominant role early in life.^166^, ^168^ Finally, immunogenic cues such as polysaccharide and secondary bile acids derived from the microbiota instruct the development of ROR𝛾t Treg cells.^169^, ^170^

Over the last decade, a growing body of evidence has indicated a role for gut dysbiosis in the pathogenesis of FA in both human subjects and animal models.^171^, ^172^ A healthy microbiota is constituted by the presence of microbial communities that programme tolerogenic responses, and conversely, FA is associated with microbial signatures that fail to curtail allergic responses to food antigens. Faecal analysis of the early‐life microbiota in babies with FA has identified a dynamic change in microbial communities associated with the loss of Clostridial species.^68^, ^173^ Administration of immunomodulatory *Clostridia* and Bacteroidetes species to FA prone mice suppressed the antigen‐specific IgE response and protected mice from anaphylaxis.^68^ This mechanism was contingent on the capacity of these bacteria to induce ROR𝛾t Treg cells, deletion of Rorc in Treg cells abrogated the protective response mediated by bacteriotherapy.^68^

In addition to the ROR𝛾t Treg cells, food antigens also drive a Th2 cell like programming in a distinct subset of Helios^+^ Treg cells in the gut mucosa. These cells express the transcription factor GATA3 and have the capacity to secrete IL4 and IL13 upon antigen exposure,^174^ shown in Figure 4C. Th2 reprogramming in Treg cells impairs their tolerogenic function by suppressing TGF‐β1 expression in a *Stat6*‐dependent manner.^69^ This pathogenic Treg subset has also been identified in human FA patients. Oral immunotherapy with omalizumab reversed the Th2 reprograming of Tregs and improved Treg cell function.^175^ Notably, ROR𝛾t^+^ Tregs and GATA3^+^ Tregs are reciprocally regulated in a balance that is largely monitored by Treg‐derived TGF‐β1. In mice, deletion of TGF‐β1 in Tregs impaired ROR𝛾t Treg cell differentiation and reciprocally expanded the pool of Th2 skewed Treg cells. Consequently, these animals had mast cell expansion in the gut and were susceptible to the development of FA.^19^, ^69^

Cross linking of high‐affinity antigen‐specific IgE present on mast cells with cognate food antigens results in mast cell degranulation and in some cases systemic anaphylaxis. Generation of antigen‐specific IgE responses is supported by Tfh cells, and a rare subset Tfh subset termed Tfh13 cells that were recently was described in mice and humans.^176^ Expressing the transcription factors BCL6 and GATA3 and characterized by high expression of the Th2 cytokines IL13, IL4 and IL5, this subset of Tfh cells controlled the induction of high‐affinity anaphylactic IgE responses to food and environmental allergens,^176^ shown in Figure 4D. The pathogenic responses of Tfh13 and IgE secreting B cells are monitored by FOXP3^+^ follicular regulatory T cells (Tfr). Tfr deficient mice are associated with higher autoantibody responses including IgG1 and IgE levels.^177^, ^178^ Ablation of Tfr cells in a mouse model of allergic house dust mite (HDM) heightened Tfh13 activity and resulted in increased production of HDM‐specific IgE and exacerbated lung inflammation.^178^ Tfr cells are endowed with the expression of the neuropeptide neuritin which represses IgE class switching and plasma cell formation of B cells.^177^ The role of Tfr cells in food allergy and the mechanism though which they control Tfh13 responses remains to be elucidated. Research over the past decade has advanced our understanding on the specific roles played by Treg cells in regulating tolerance to food antigens. Distinct subsets of Tregs and Tfh cells have now been identified that are implicated in the pathophysiology of FA. Future studies translating this knowledge to clinics will be pertinent in quelling the rise of FA.

## 7 TREG CELLS IN ASTHMA

Asthma is a heterogenous chronic inflammatory condition of the airways affecting up to 30% of the population in different countries. It is characterized by diverse respiratory symptoms including cough, wheeze, shortness of breath and chest tightness; which are driven by the inflammation of the airways and trigger processes such as mucus production, airway remodelling and bronchial hyperreactivity.^179^ Asthma manifests as different phenotypes and endotypes with specific etiopathogenic mechanisms, broadly classified as type‐2 immune‐mediated or non‐type 2 immune‐mediated, and based on blood and tissue eosinophilia, exhaled nitric oxide levels and total and specific IgE.^180^ Allergic / type‐2 asthma is usually triggered by sensitization in early life to environmental allergens such as HDM, pollen, animal dander or cockroach.^181^ Upon recognition of these triggers, allergen‐specific Th2 cells release type‐2 cytokines (IL‐4, IL‐5, IL‐9, IL‐13) that lead to airway eosinophilia, mucus oversecretion and priming of B cells for allergen‐specific IgE synthesis.

Treg dysfunction plays an essential role in asthma pathogenesis, as these cells are key regulators of tolerance mechanisms. In the lung, immune tolerance is mainly controlled by three Treg subsets: FOXP3 iTregs, T regulatory 1 (Tr1) cells and follicular Tregs (Tfr).^182^ These cell types promote the differentiation of regulatory B cells and prime DC towards a tolerogenic profile, hence inhibiting the proximal pathways of sensitization and IgE production triggered upon allergen exposure.^183^ By producing IL‐10, IL‐35, TGF‐β, CTLA‐4, PD1 and other tolerogenic mediators, Treg subsets in the lung restrain the function of Th2 cells, eosinophils, ILC2s, mast cells and basophils, the most essential cells involved in the allergic asthma reaction.^182^ Early studies in murine allergic asthma models have shown that depletion of CD4 + CD25+ Tregs enhances neutrophil and T‐cell recruitment to the airways, IL‐4 and IL‐5 production, and airway hyperreactivity.^184^

Patients with severe asthma show reduced numbers of FOXP3+ Tregs in the bronchoalveolar fluid compared to healthy controls, and decreased levels of circulating Tregs with impaired migration to the lung epithelium.^185^, ^186^, ^187^ In these patients, FOXP3+ Tregs display reduced expression levels of CCR5, indicating an impaired suppressive activity correlating with worsened lung function parameters.^185^ In addition, Tregs in asthma show high expression levels of CRTH2, a type 2 receptor for prostaglandin D2, associated with asthma control and exacerbation.^188^ Thus, allergic asthma is characterized by decreased Treg numbers, with low CCR5 and high CRTH2 expression indicating an impaired functionality and a Th2‐biased phenotype.

### 7.1 Intrinsic and extrinsic influences on Treg function affecting asthma development and exacerbation

A strong genetic component underlies asthma susceptibility, as shown by multiple studies (reviewed in^189^). With recent advances in sequencing technologies, a plethora of genome‐wide association studies (GWAS) have identified various loci associated with asthma,^190^ including several single nucleotide polymorphisms (SNPs) associated with the type‐2 endotype.^191^, ^192^ Recently, a large meta‐analysis combining GWAS results from Iceland and UK biobanks reported 88 independent associations at 56 loci, the majority involved in the regulation of CD4+ T‐cell activation, responses and physiology.^193^ Regarding Tregs, it is known that monogenic mutations affecting FOXP3 (i.e. IPEX syndrome in humans or scurfy mice) cause severe immune dysregulation with autoimmunity and allergic manifestations, including elevated serum IgE and peripheral eosinophilia.^194^ Another loci controlling Treg function which shows a strong association with asthma is the Ryanodine receptor 2 (RyR2),^195^ a calcium channel that mediates the contractile response in airway smooth muscle cells.^196^, ^197^ Recently, it was shown that FOXP3 blocks the expression of RyR2 in Tregs, reducing the m‐calpain activity necessary for their disengagement from DC, thereby enhancing contact‐dependent suppressive activity.^198^ Interestingly, the authors showed that shRNA‐mediated depletion of RyR2 in conventional CD4+ T cells strengthened their interaction with DC, rendering them immunosuppressive and making them capable of improving ovalbumin‐induced airway inflammation and autoimmunity in Scurfy mice.^198^

**FIGURE 5:**
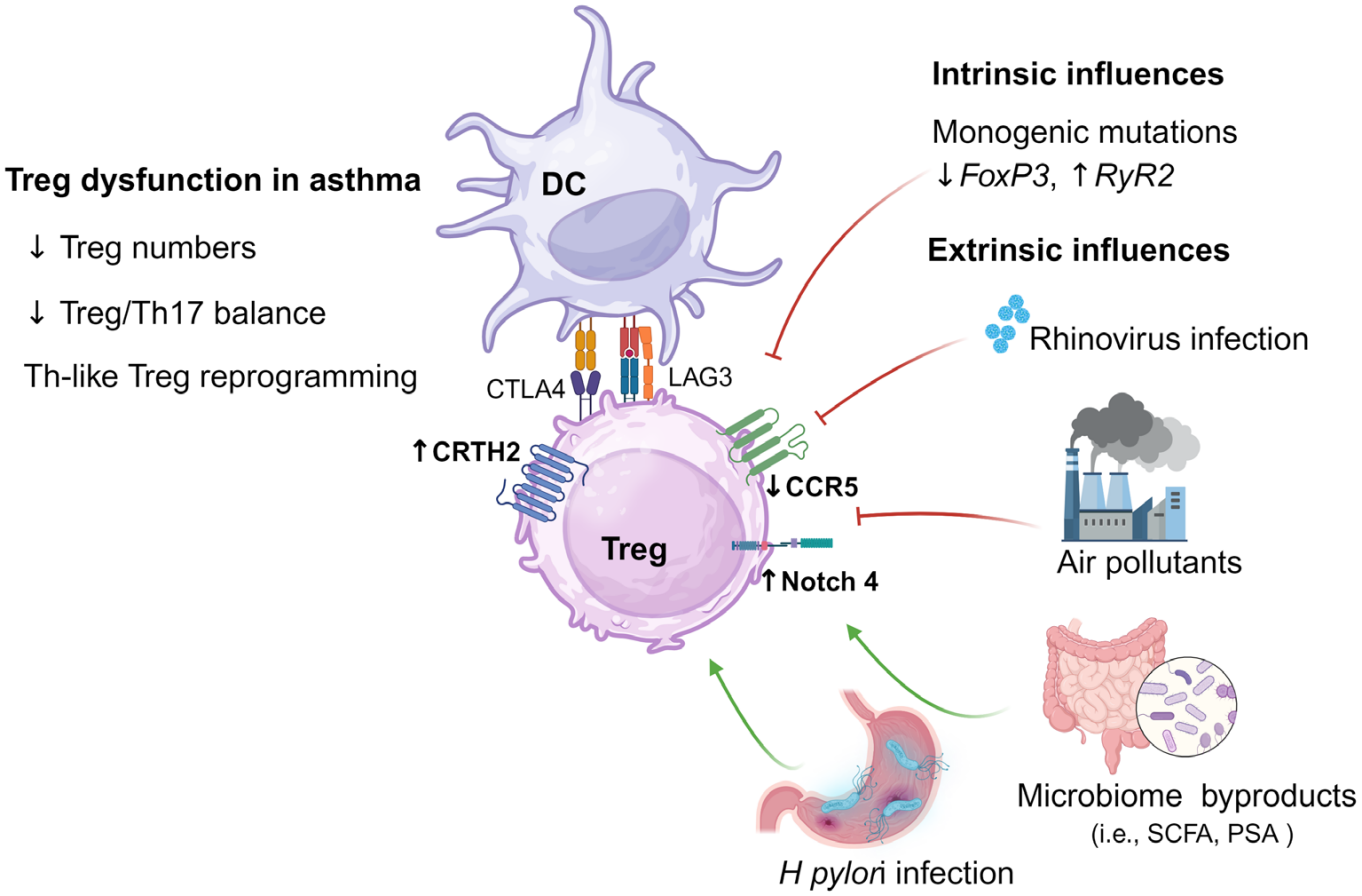
Intrinsic and extrinsic factors contributing to Treg dysfunction in asthma. Asthma patients exhibit decreased Treg counts and Treg/Th17 balance as well as reprogramming of Tregs towards a Th2‐like phenotype. Monogenic mutations and SNPs, as revealed by GWAS studies, represent the main intrinsic factor influencing Treg function in asthma. As for extrinsic influences, rhinoviral infections and environmental pollutants are important negative modulators of Treg function contributing to asthma exacerbations. On the other hand, microbial byproducts derived from gut commensals and also pathogenic bacteria (i.e. *H*. *pylori*) are important Treg inducers with potential therapeutic application.

The influence of environmental factors on asthma exacerbations is well established. Among such factors, rhinoviral infections are strongly associated with severe exacerbations.^199^ Rhinovirus infection has been shown to directly affect the suppressive activity of Tregs, rendering them less able to inhibit type 2 immune responses,^200^ shown in Figure 5. Another important risk factor responsible for asthma exacerbation is exposure to high levels of ambient air pollution,^201^ which is associated with epigenetic changes affecting Treg function. Common environmental pollutants (i.e. polycyclic aromatic hydrocarbons (PAHs), CO, NO_2_ and particulate matter (PM)) induce alterations in CpG methylation of the FOXP3 locus, impairing Treg activity and worsening the asthma phenotype.^201^, ^202^ Further studies indicate a strong correlation between childhood exposure to air pollutants and FOXP3 methylation levels, further associated with Treg dysfunction and increased plasma IgE levels,^203^ Figure 5. Additionally, PM exposure has been shown to alter the Treg/Th17 cell balance, aggravating asthma manifestations in an aryl hydrocarbon receptor (Ahr)‐dependent manner.^204^ PM‐triggered activation of Ahr, in turn, induces the expression of the Notch ligand JAG1, driving iTreg destabilization and promoting allergic airway inflammation.^11^, ^205^ In recent studies, we have identified Notch4 as the receptor on Tregs involved in these interactions, which is upregulated in circulating Tregs of asthma subjects in an IL‐6‐dependent manner.^72^, ^206^ Interestingly, targeting IL‐6R signalling can enhance the immunosuppressive properties of Tregs in association with inhibition of Notch4 expression, which may represent a therapeutic opportunity for patients with severe asthma.^207^, ^208^, ^209^

Finally, the important role of the microbiome in shaping immune responses along the gut–lung axis is being increasingly recognized, as recently reviewed in.^210^ Gut commensal bacteria can control T‐cell homeostasis by modulating the Treg/Th17 cell balance,^21^ shown in Figure 5. For instance, *Bacteroides fragilis* and several *Clostridium* strains are known inducers of Tregs, driven by microbial byproducts like *Bacteroides*‐derived polysaccharide A (PSA)^211^ or short chain fatty acids (SCFA).^212^, ^213^ Butyrate and propionate, in particular, are strong Treg inducers through the inhibition of histone deacetylases that affect the FOXP3 promoter, thereby promoting Treg induction and stability.^214^, ^215^ Indeed, oral administration of PSA induced IL‐10‐producing Tregs and attenuated the asthmatic phenotype in a murine model, suggesting that these bacterial metabolites may provide opportunities for therapeutic interventions.^216^

Another important Treg inducer in the gastrointestinal tract is the gram‐negative bacteria *Helicobacter pylori*, which currently affects approximately 50% of the world population.^217^ Interestingly, several epidemiological studies highlight the beneficial role of this—once commensal—bacteria in the development of asthma.^218^, ^219^, ^220^ This association likely stems from the Treg inducing ability of *H*. *Pylori*,^221^, ^222^ which depends on bacterial virulence factors like cytotoxin‐associated gene A (CagA), vacuolating cytotoxin A (VacA) and γ‐glutamyl transferase (GGT).^223^ More recently, animal studies confirmed the epidemiological data by showing that neonatal infection with *H. pylori* led to an attenuated asthma phenotype in later life through the induction of Tregs.^224^ Further studies showed that *H. pylori* reprograms DC towards a tolerogenic IL‐18‐producing phenotype that promotes the development of immunoprotective Tregs.^225^ Therapeutic applications of *H. pylori* in asthma have also been envisioned, using bacterial extracts or purified virulence proteins. For instance, prophylactic application of *H*. *pylori*‐derived extracts modulated DC and Treg responses via IL‐10 signalling, attenuating allergic airway disease in later life.^226^ Likewise, recent animal studies highlighted the potential prophylactic and therapeutic effects of applying purified VacA in acute and chronic allergic airway disease.^226^, ^227^, ^228^, ^229^

## 8 CONCLUSION

In the last decade, we have deepened our knowledge of the immune mechanisms driving allergic diseases and their treatment. Tolerance to environmental and food allergens is core to a healthy immune response, and in this context, allergen‐specific Tregs are crucial players in the prevention of allergic diseases and for successful immunotherapy. To date, allergen immunotherapy remains the only intervention with the potential to restore immune function in allergic diseases, being the subject of intensive research to provide mechanistic insights into its protective effects. Treg dysfunction is central to the pathogenesis of allergic disorders and is characterized by loss of peripheral tolerance and the generation of chronic inflammation. Hence, a better understanding of the mechanisms driving FOXP3 expression and Treg stabilization will contribute to the development of novel therapeutic strategies for these disorders.

## AUTHOR CONTRIBUTIONS

M.L.C and G.B wrote the asthma section. S.M and M. D wrote the skin section. E.S.V wrote the food allergy section. X.C and H.H wrote the introduction and the main Treg section. M.L.C and H.H revised, edited and finalized the manuscript.

## 10 FUNDING INFORMATION

Deutsche Forschungsgemeinschaft, Grant/Award Number: CO 1058/3–2 (Melanie L. Conrad).

## CONFLICT OF INTEREST STATEMENT

All authors declare that there was no conflict of interest when writing this manuscript.

## Data Availability

Data sharing not applicable to this article as no datasets were generated or analysed during the current study.

## References

[1] Pfefferle PI , Keber CU , Cohen RM , Garn H . The hygiene hypothesis ‐ learning from but not living in the past. Front Immunol. 2021;12:635935. 33796103 10.3389/fimmu.2021.635935 PMC8007786

[2] Lambrecht BN , Hammad H . The immunology of the allergy epidemic and the hygiene hypothesis. Nat Immunol. 2017;18(10):1076‐1083. 28926539 10.1038/ni.3829

[3] Yazdanbakhsh M , Kremsner PG , van Ree R . Allergy, parasites, and the hygiene hypothesis. Science. 2002;296(5567):490‐494. 11964470 10.1126/science.296.5567.490

[4] Donald K , Finlay BB . Early‐life interactions between the microbiota and immune system: impact on immune system development and atopic disease. Nat Rev Immunol. 2023;23(11):735‐748. 37138015 10.1038/s41577-023-00874-w

[5] Ege MJ . The hygiene hypothesis in the age of the microbiome. Ann Am Thorac Soc. 2017;14(Supplement_5):S348‐S353. 29161087 10.1513/AnnalsATS.201702-139AW

[6] Gupta RS , Warren CM , Smith BM , et al. Prevalence and severity of food allergies among US adults. JAMA Netw Open. 2019;2(1):e185630. 30646188 10.1001/jamanetworkopen.2018.5630 PMC6324316

[7] Hill DA , Spergel JM . The atopic march: critical evidence and clinical relevance. Ann Allergy Asthma Immunol. 2018;120(2):131‐137. 29413336 10.1016/j.anai.2017.10.037 PMC5806141

[8] Sanchez‐Borges M , Martin BL , Muraro AM , et al. The importance of allergic disease in public health: an iCAALL statement. World Allergy Organ J. 2018;11(1):8. 29743965 10.1186/s40413-018-0187-2 PMC5921992

[9] Fujimura KE , Lynch SV . Microbiota in allergy and asthma and the emerging relationship with the gut microbiome. Cell Host Microbe. 2015;17(5):592‐602. 25974301 10.1016/j.chom.2015.04.007 PMC4443817

[10] Barrientos G , Ronchi F , Conrad ML . Nutrition during pregnancy: influence on the gut microbiome and fetal development. Am J Reprod Immunol. 2024;91(1):e13802. 38282608 10.1111/aji.13802

[11] Xia M , Harb H , Saffari A , Sioutas C , Chatila TA . A jagged 1‐notch 4 molecular switch mediates airway inflammation induced by ultrafine particles. J Allergy Clin Immunol. 2018;142(4):1243‐1256. 29627423 10.1016/j.jaci.2018.03.009 PMC6173656

[12] Hagner S , Harb H , Zhao M , et al. Farm‐derived gram‐positive bacterium Staphylococcus sciuri W620 prevents asthma phenotype in HDM‐ and OVA‐exposed mice. Allergy. 2013;68(3):322‐329. 23369007 10.1111/all.12094

[13] Conrad ML , Ferstl R , Teich R , et al. Maternal TLR signaling is required for prenatal asthma protection by the nonpathogenic microbe Acinetobacter lwoffii F78. J Exp Med. 2009;206(13):2869‐2877. 19995952 10.1084/jem.20090845 PMC2806458

[14] Cait A , Wedel A , Arntz JL , et al. Prenatal antibiotic exposure, asthma, and the atopic march: a systematic review and meta‐analysis. Allergy. 2022;77(11):3233‐3248. 35689800 10.1111/all.15404

[15] Zhang Z , Wang J , Wang H , et al. Association of infant antibiotic exposure and risk of childhood asthma: a meta‐analysis. World Allergy Organ J. 2021;14(11):100607. 34934469 10.1016/j.waojou.2021.100607 PMC8661061

[16] Alhasan MM , Cait AM , Heimesaat MM , et al. Antibiotic use during pregnancy increases offspring asthma severity in a dose‐dependent manner. Allergy. 2020;75(8):1979‐1990. 32064643 10.1111/all.14234

[17] Harb H , Amarasekera M , Ashley S , et al. Epigenetic regulation in early childhood: a miniaturized and validated method to assess histone acetylation. Int Arch Allergy Immunol. 2015;168(3):173‐181. 26789836 10.1159/000442158

[18] Hong X , Wang X . Early life precursors, epigenetics, and the development of food allergy. Semin Immunopathol. 2012;34(5):655‐669. 22777545 10.1007/s00281-012-0323-y PMC3439840

[19] Stephen‐Victor E , Crestani E , Chatila TA . Dietary and microbial determinants in food allergy. Immunity. 2020;53(2):277‐289. 32814026 10.1016/j.immuni.2020.07.025 PMC7467210

[20] Harb H , van Tol EA , Heine H , et al. Neonatal supplementation of processed supernatant from lactobacillus rhamnosus GG improves allergic airway inflammation in mice later in life. Clin Exp Allergy. 2013;43(3):353‐364. 23414544 10.1111/cea.12047

[21] Alhasan MM , Holsken O , Duerr C , et al. Antibiotic use during pregnancy is linked to offspring gut microbial dysbiosis, barrier disruption, and altered immunity along the gut‐lung axis. Eur J Immunol. 2023;53(10):e2350394. 37431194 10.1002/eji.202350394

[22] Catak MC , Akcam B , Bilgic Eltan S , et al. Comparing the levels of CTLA‐4‐dependent biological defects in patients with LRBA deficiency and CTLA‐4 insufficiency. Allergy. 2022;77(10):3108‐3123. 35491430 10.1111/all.15331

[23] Gao YD , Xepapadaki P , Cui YW , et al. Effect of Haemophilus influenzae, Streptococcus pneumoniae and influenza vaccinations on infections, immune response and asthma control in preschool children with asthma. Allergy. 2023;78(6):1473‐1488. 36229409 10.1111/all.15551

[24] Chatila TA , Blaeser F , Ho N , et al. JM2, encoding a fork head‐related protein, is mutated in X‐linked autoimmunity‐allergic disregulation syndrome. J Clin Invest. 2000;106(12):R75‐R81. 11120765 10.1172/JCI11679 PMC387260

[25] Palomares O , Martin‐Fontecha M , Lauener R , et al. Regulatory T cells and immune regulation of allergic diseases: roles of IL‐10 and TGF‐beta. Genes Immun. 2014;15(8):511‐520. 25056447 10.1038/gene.2014.45

[26] Bilate AM , Lafaille JJ . Induced CD4+Foxp3+ regulatory T cells in immune tolerance. Annu Rev Immunol. 2012;30:733‐758. 22224762 10.1146/annurev-immunol-020711-075043

[27] Lio CW , Hsieh CS . Becoming self‐aware: the thymic education of regulatory T cells. Curr Opin Immunol. 2011;23(2):213‐219. 21146972 10.1016/j.coi.2010.11.010 PMC3061250

[28] Chang L , Wu H , Huang W , et al. IL‐21 induces pyroptosis of Treg cells via Akt‐mTOR‐NLRP3‐caspase 1 axis in eosinophilic chronic rhinosinusitis. J Allergy Clin Immunol. 2023;152(3):641‐655. 37164271 10.1016/j.jaci.2023.04.013

[29] Chatila TA . Regulatory T cells: key players in tolerance and autoimmunity. Endocrinol Metab Clin N Am. 2009;38(2):265‐272, vii. 10.1016/j.ecl.2009.01.002 PMC2700045 19328410

[30] Davidson TS , DiPaolo RJ , Andersson J , Shevach EM . Cutting edge: IL‐2 is essential for TGF‐beta‐mediated induction of Foxp3+ T regulatory cells. J Immunol. 2007;178(7):4022‐4026. 17371955 10.4049/jimmunol.178.7.4022

[31] Xiao S , Jin H , Korn T , et al. Retinoic acid increases Foxp3+ regulatory T cells and inhibits development of Th17 cells by enhancing TGF‐beta‐driven Smad3 signaling and inhibiting IL‐6 and IL‐23 receptor expression. J Immunol. 2008;181(4):2277‐2284. 18684916 10.4049/jimmunol.181.4.2277 PMC2722959

[32] Hong JY , Li SS , Hu TY , et al. Frontline science: TLR3 activation inhibits food allergy in mice by inducing IFN‐gamma(+) Foxp3(+) regulatory T cells. J Leukoc Biol. 2019;106(6):1201‐1209. 30997942 10.1002/JLB.3HI0918-348RR

[33] Xu LZ , Xie RD , Xie H , et al. Chimeric specific antigen epitope‐carrying dendritic cells induce interleukin‐17(+) regulatory T cells to suppress food allergy. Clin Exp Allergy. 2020;50(2):231‐243. 31715648 10.1111/cea.13528

[34] Haribhai D , Lin W , Edwards B , et al. A central role for induced regulatory T cells in tolerance induction in experimental colitis. J Immunol. 2009;182(6):3461‐3468. 19265124 10.4049/jimmunol.0802535 PMC2763205

[35] Kitani A , Fuss I , Nakamura K , Kumaki F , Usui T , Strober W . Transforming growth factor (TGF)‐beta1‐producing regulatory T cells induce Smad‐mediated interleukin 10 secretion that facilitates coordinated immunoregulatory activity and amelioration of TGF‐beta1‐mediated fibrosis. J Exp Med. 2003;198(8):1179‐1188. 14557415 10.1084/jem.20030917 PMC2194234

[36] Collison LW , Chaturvedi V , Henderson AL , et al. IL‐35‐mediated induction of a potent regulatory T cell population. Nat Immunol. 2010;11(12):1093‐1101. 20953201 10.1038/ni.1952 PMC3008395

[37] Grossman WJ , Verbsky JW , Barchet W , Colonna M , Atkinson JP , Ley TJ . Human T regulatory cells can use the perforin pathway to cause autologous target cell death. Immunity. 2004;21(4):589‐601. 15485635 10.1016/j.immuni.2004.09.002

[38] Gondek DC , Lu LF , Quezada SA , Sakaguchi S , Noelle RJ . Cutting edge: contact‐mediated suppression by CD4+CD25+ regulatory cells involves a granzyme B‐dependent, perforin‐independent mechanism. J Immunol. 2005;174(4):1783‐1786. 15699103 10.4049/jimmunol.174.4.1783

[39] Sula Karreci E , Eskandari SK , Dotiwala F , et al. Human regulatory T cells undergo self‐inflicted damage via granzyme pathways upon activation. JCI Insight. 2017;2(21):e91599. 29093262 10.1172/jci.insight.91599 PMC5690280

[40] Thornton AM , Shevach EM . CD4+CD25+ immunoregulatory T cells suppress polyclonal T cell activation in vitro by inhibiting interleukin 2 production. J Exp Med. 1998;188(2):287‐296. 9670041 10.1084/jem.188.2.287 PMC2212461

[41] Kim HJ , Barnitz RA , Kreslavsky T , et al. Stable inhibitory activity of regulatory T cells requires the transcription factor Helios. Science. 2015;350(6258):334‐339. 26472910 10.1126/science.aad0616 PMC4627635

[42] Read S , Malmstrom V , Powrie F . Cytotoxic T lymphocyte‐associated antigen 4 plays an essential role in the function of CD25(+)CD4(+) regulatory cells that control intestinal inflammation. J Exp Med. 2000;192(2):295‐302. 10899916 10.1084/jem.192.2.295 PMC2193261

[43] Ruffo E , Wu RC , Bruno TC , Workman CJ , Vignali DAA . Lymphocyte‐activation gene 3 (LAG3): the next immune checkpoint receptor. Semin Immunol. 2019;42:101305. 31604537 10.1016/j.smim.2019.101305 PMC6920665

[44] Liang B , Workman C , Lee J , et al. Regulatory T cells inhibit dendritic cells by lymphocyte activation gene‐3 engagement of MHC class II. J Immunol. 2008;180(9):5916‐5926. 18424711 10.4049/jimmunol.180.9.5916

[45] Deaglio S , Dwyer KM , Gao W , et al. Adenosine generation catalyzed by CD39 and CD73 expressed on regulatory T cells mediates immune suppression. J Exp Med. 2007;204(6):1257‐1265. 17502665 10.1084/jem.20062512 PMC2118603

[46] Schiering C , Krausgruber T , Chomka A , et al. The alarmin IL‐33 promotes regulatory T‐cell function in the intestine. Nature. 2014;513(7519):564‐568. 25043027 10.1038/nature13577 PMC4339042

[47] Wu J , Ren B , Wang D , Lin H . Regulatory T cells in skeletal muscle repair and regeneration: recent insights. Cell Death Dis. 2022;13(8):680. 35931697 10.1038/s41419-022-05142-8 PMC9356005

[48] Koga R , Maehara T , Aoyagi R , et al. Granzyme K‐ and amphiregulin‐expressing cytotoxic T cells and activated extrafollicular B cells are potential drivers of IgG4‐related disease. J Allergy Clin Immunol. 2024;153(4):1095‐1112. 38092138 10.1016/j.jaci.2023.11.916

[49] Sidoti Migliore G , Campana S , Barberi C , et al. Mechanical bacterial lysate enhances antimicrobial barrier mechanisms in human airway epithelial cells. J Leukoc Biol. 2023;113(5):535‐540. 36807710 10.1093/jleuko/qiad003

[50] Beuraud C , Lombardi V , Luce S , et al. CCR10(+) ILC2s with ILC1‐like properties exhibit a protective function in severe allergic asthma. Allergy. 2019;74(5):933‐943. 30475388 10.1111/all.13679

[51] Harb H , Benamar M , Lai PS , et al. Notch4 signaling limits regulatory T‐cell‐mediated tissue repair and promotes severe lung inflammation in viral infections. Immunity. 2021;54(6):1186‐1199. 33915108 10.1016/j.immuni.2021.04.002 PMC8080416

[52] Scheffschick A , Kiritsi D , Magin TM . Keratin defects trigger the itch‐inducing cytokine thymic stromal lymphopoietin through amphiregulin‐epidermal growth factor receptor signaling. J Allergy Clin Immunol. 2019;144(6):1719‐1722. 31425777 10.1016/j.jaci.2019.07.041

[53] Azumi J , Takeda T , Shimada Y , et al. Organogermanium THGP induces differentiation into M1 macrophages and suppresses the proliferation of melanoma cells via phagocytosis. Int J Mol Sci. 2023;24(3):1885. 36768216 10.3390/ijms24031885 PMC9915250

[54] Guan T , Zhou X , Zhou W , Lin H . Regulatory T cell and macrophage crosstalk in acute lung injury: future perspectives. Cell Death Dis. 2023;9(1):9. 10.1038/s41420-023-01310-7 PMC9841501 36646692

[55] Li J , Tan J , Martino MM , Lui KO . Regulatory T‐cells: potential regulator of tissue repair and regeneration. Front Immunol. 2018;9:585. 29662491 10.3389/fimmu.2018.00585 PMC5890151

[56] Ward ST , Li KK , Hepburn E , et al. The effects of CCR5 inhibition on regulatory T‐cell recruitment to colorectal cancer. Br J Cancer. 2015;112(2):319‐328. 25405854 10.1038/bjc.2014.572 PMC4301825

[57] Tan MC , Goedegebuure PS , Belt BA , et al. Disruption of CCR5‐dependent homing of regulatory T cells inhibits tumor growth in a murine model of pancreatic cancer. J Immunol. 2009;182(3):1746‐1755. 19155524 10.4049/jimmunol.182.3.1746 PMC3738070

[58] Yao Y , Chen CL , Yu D , Liu Z . Roles of follicular helper and regulatory T cells in allergic diseases and allergen immunotherapy. Allergy. 2021;76(2):456‐470. 33098663 10.1111/all.14639

[59] Jutel M , Akdis M , Blaser K , Akdis CA . Mechanisms of allergen specific immunotherapy—T‐cell tolerance and more. Allergy. 2006;61(7):796‐807. 16792576 10.1111/j.1398-9995.2006.01175.x

[60] Roth‐Walter F , Adcock IM , Benito‐Villalvilla C , et al. Immune modulation via T regulatory cell enhancement: disease‐modifying therapies for autoimmunity and their potential for chronic allergic and inflammatory diseases‐an EAACI position paper of the task force on Immunopharmacology (TIPCO). Allergy. 2021;76(1):90‐113. 32593226 10.1111/all.14478

[61] Sage PT , Sharpe AH . T follicular regulatory cells. Immunol Rev. 2016;271(1):246‐259. 27088919 10.1111/imr.12411

[62] Sage PT , Tan CL , Freeman GJ , Haigis M , Sharpe AH . Defective TFH cell function and increased TFR cells contribute to defective antibody production in aging. Cell Rep. 2015;12(2):163‐171. 26146074 10.1016/j.celrep.2015.06.015 PMC4504745

[63] Tsuji M , Komatsu N , Kawamoto S , et al. Preferential generation of follicular B helper T cells from Foxp3+ T cells in gut Peyer's patches. Science. 2009;323(5920):1488‐1492. 19286559 10.1126/science.1169152

[64] Koch MA , Tucker‐Heard G , Perdue NR , Killebrew JR , Urdahl KB , Campbell DJ . The transcription factor T‐bet controls regulatory T cell homeostasis and function during type 1 inflammation. Nat Immunol. 2009;10(6):595‐602. 19412181 10.1038/ni.1731 PMC2712126

[65] Chung Y , Tanaka S , Chu F , et al. Follicular regulatory T cells expressing Foxp3 and Bcl‐6 suppress germinal center reactions. Nat Med. 2011;17(8):983‐988. 21785430 10.1038/nm.2426 PMC3151340

[66] Trujillo‐Ochoa JL , Kazemian M , Afzali B . The role of transcription factors in shaping regulatory T cell identity. Nat Rev Immunol. 2023;23(12):842‐856. 37336954 10.1038/s41577-023-00893-7 PMC10893967

[67] Dolsten GA , Pritykin Y . Genomic analysis of Foxp3 function in regulatory T cells. J Immunol. 2023;210(7):880‐887. 36947819 10.4049/jimmunol.2200864 PMC10037560

[68] Abdel‐Gadir A , Stephen‐Victor E , Gerber GK , et al. Microbiota therapy acts via a regulatory T cell MyD88/RORgammat pathway to suppress food allergy. Nat Med. 2019;25(7):1164‐1174. 31235962 10.1038/s41591-019-0461-z PMC6677395

[69] Turner JA , Stephen‐Victor E , Wang S , et al. Regulatory T cell‐derived TGF‐beta1 controls multiple checkpoints governing allergy and autoimmunity. Immunity. 2020;53(6):1202‐1214. 33086036 10.1016/j.immuni.2020.10.002 PMC7744401

[70] Tao B , Ruan G , Wang D , Li Y , Wang Z , Yin G . Imbalance of peripheral Th17 and regulatory T cells in children with allergic rhinitis and bronchial asthma. Iran J Allergy Asthma Immunol. 2015;14(3):273‐279. 26546895

[71] Voo KS , Wang YH , Santori FR , et al. Identification of IL‐17‐producing FOXP3+ regulatory T cells in humans. Proc Natl Acad Sci USA. 2009;106(12):4793‐4798. 19273860 10.1073/pnas.0900408106 PMC2653560

[72] Harb H , Stephen‐Victor E , Crestani E , et al. A regulatory T cell Notch4‐GDF15 axis licenses tissue inflammation in asthma. Nat Immunol. 2020;21:1359‐1370. 32929274 10.1038/s41590-020-0777-3 PMC7578174

[73] Massoud AH , Charbonnier LM , Lopez D , Pellegrini M , Phipatanakul W , Chatila TA . An asthma‐associated IL4R variant exacerbates airway inflammation by promoting conversion of regulatory T cells to TH17‐like cells. Nat Med. 2016;22(9):1013‐1022. 27479084 10.1038/nm.4147 PMC5014738

[74] Casaca VI , Illi S , Klucker E , et al. STAT6 polymorphisms are associated with neonatal regulatory T cells and cytokines and atopic diseases at 3 years. Allergy. 2013;68(10):1249‐1258. 24053457 10.1111/all.12220

[75] Malmhall C , Bossios A , Pullerits T , Lotvall J . Effects of pollen and nasal glucocorticoid on FOXP3+, GATA‐3+ and T‐bet+ cells in allergic rhinitis. Allergy. 2007;62(9):1007‐1013. 17686103 10.1111/j.1398-9995.2007.01420.x

[76] Leonard C , Montamat G , Davril C , et al. Comprehensive mapping of immune tolerance yields a regulatory TNF receptor 2 signature in a murine model of successful Fel d 1‐specific immunotherapy using high‐dose CpG adjuvant. Allergy. 2021;76(7):2153‐2165. 33345329 10.1111/all.14716 PMC8359185

[77] Saxena V , Lakhan R , Iyyathurai J , Bromberg JS . Mechanisms of exTreg induction. Eur J Immunol. 2021;51(8):1956‐1967. 33975379 10.1002/eji.202049123 PMC8338747

[78] Joudi AM , Reyes Flores CP , Singer BD . Epigenetic control of regulatory T cell stability and function: implications for translation. Front Immunol. 2022;13:861607. 35309306 10.3389/fimmu.2022.861607 PMC8924620

[79] Kawakami R , Kitagawa Y , Chen KY , et al. Distinct Foxp3 enhancer elements coordinate development, maintenance, and function of regulatory T cells. Immunity. 2021;54(5):947‐961. 33930308 10.1016/j.immuni.2021.04.005

[80] Wang L , Liu Y , Beier UH , et al. Foxp3+ T‐regulatory cells require DNA methyltransferase 1 expression to prevent development of lethal autoimmunity. Blood. 2013;121(18):3631‐3639. 23444399 10.1182/blood-2012-08-451765 PMC3643763

[81] Liu Y , Wang L , Han R , et al. Two histone/protein acetyltransferases, CBP and p300, are indispensable for Foxp3+ T‐regulatory cell development and function. Mol Cell Biol. 2014;34(21):3993‐4007. 25154413 10.1128/MCB.00919-14 PMC4386456

[82] Yang R , Qu C , Zhou Y , et al. Hydrogen sulfide promotes Tet1‐ and Tet2‐mediated Foxp3 demethylation to drive regulatory T cell differentiation and maintain immune homeostasis. Immunity. 2015;43(2):251‐263. 26275994 10.1016/j.immuni.2015.07.017 PMC4731232

[83] Li Y , Deuring J , Peppelenbosch MP , Kuipers EJ , de Haar C , van der Woude CJ . IL‐6‐induced DNMT1 activity mediates SOCS3 promoter hypermethylation in ulcerative colitis‐related colorectal cancer. Carcinogenesis. 2012;33(10):1889‐1896. 22739025 10.1093/carcin/bgs214

[84] Akdis CA . Does the epithelial barrier hypothesis explain the increase in allergy, autoimmunity and other chronic conditions? Nat Rev Immunol. 2021;21(11):739‐751. 33846604 10.1038/s41577-021-00538-7

[85] Celebi Sozener Z , Ozdel Ozturk B , Cerci P , et al. Epithelial barrier hypothesis: effect of the external exposome on the microbiome and epithelial barriers in allergic disease. Allergy. 2022;77(5):1418‐1449. 35108405 10.1111/all.15240 PMC9306534

[86] Yazici D , Ogulur I , Pat Y , et al. The epithelial barrier: the gateway to allergic, autoimmune, and metabolic diseases and chronic neuropsychiatric conditions. Semin Immunol. 2023;70:101846. 37801907 10.1016/j.smim.2023.101846

[87] Gallo RL . Human skin is the largest epithelial surface for interaction with microbes. J Invest Dermatol. 2017;137(6):1213‐1214. 28395897 10.1016/j.jid.2016.11.045 PMC5814118

[88] Li R , Li J , Zhou X . Lung microbiome: new insights into the pathogenesis of respiratory diseases. Signal Transduct Target Ther. 2024;9(1):19. 38228603 10.1038/s41392-023-01722-y PMC10791971

[89] Rath E , Haller D . Intestinal epithelial cell metabolism at the interface of microbial dysbiosis and tissue injury. Mucosal Immunol. 2022;15(4):595‐604. 35534699 10.1038/s41385-022-00514-x PMC9259489

[90] Heijink IH , Kuchibhotla VNS , Roffel MP , et al. Epithelial cell dysfunction, a major driver of asthma development. Allergy. 2020;75(8):1902‐1917. 32460363 10.1111/all.14421 PMC7496351

[91] Kim BE , Leung DYM . Significance of skin barrier dysfunction in atopic dermatitis. Allergy, Asthma Immunol Res. 2018;10(3):207‐215. 29676067 10.4168/aair.2018.10.3.207 PMC5911439

[92] Leung DYM , Berdyshev E , Goleva E . Cutaneous barrier dysfunction in allergic diseases. J Allergy Clin Immunol. 2020;145(6):1485‐1497. 32507227 10.1016/j.jaci.2020.02.021 PMC7291847

[93] Huang ZQ , Liu J , Sun LY , et al. Updated epithelial barrier dysfunction in chronic rhinosinusitis: targeting pathophysiology and treatment response of tight junctions. Allergy. 2024;79(5):1146‐1165. 38372149 10.1111/all.16064

[94] McGowan EC , Singh R , Katzka DA . Barrier dysfunction in eosinophilic esophagitis. Curr Gastroenterol Rep. 2023;25(12):380‐389. 37950816 10.1007/s11894-023-00904-6 PMC12869379

[95] Knoedler S , Knoedler L , Kauke‐Navarro M , et al. Regulatory T cells in skin regeneration and wound healing. Mil Med Res. 2023;10(1):49. 37867188 10.1186/s40779-023-00484-6 PMC10591349

[96] Chaudhry A , Samstein RM , Treuting P , et al. Interleukin‐10 signaling in regulatory T cells is required for suppression of Th17 cell‐mediated inflammation. Immunity. 2011;34(4):566‐578. 21511185 10.1016/j.immuni.2011.03.018 PMC3088485

[97] Hu J , Kang H , Liu C , Hu P , Yang M , Zhou F . Regulatory T cells could improve intestinal barrier dysfunction in heatstroke. Inflammation. 2019;42(4):1228‐1238. 30820807 10.1007/s10753-019-00983-6

[98] Bae MJ , Shin HS , See HJ , Jung SY , Kwon DA , Shon DH . Baicalein induces CD4(+)Foxp3(+) T cells and enhances intestinal barrier function in a mouse model of food allergy. Sci Rep. 2016;6:32225. 27561877 10.1038/srep32225 PMC4999817

[99] Mock JR , Garibaldi BT , Aggarwal NR , et al. Foxp3+ regulatory T cells promote lung epithelial proliferation. Mucosal Immunol. 2014;7(6):1440‐1451. 24850425 10.1038/mi.2014.33 PMC4205163

[100] Werfel T , Allam JP , Biedermann T , et al. Cellular and molecular immunologic mechanisms in patients with atopic dermatitis. J Allergy Clin Immunol. 2016;138(2):336‐349. 27497276 10.1016/j.jaci.2016.06.010

[101] Tan BB , Weald D , Strickland I , Friedmann PS . Double‐blind controlled trial of effect of housedust‐mite allergen avoidance on atopic dermatitis. Lancet. 1996;347(8993):15‐18. 8531541 10.1016/s0140-6736(96)91556-1

[102] Schuler CF , Tsoi LC , Billi AC , Harms PW , Weidinger S , Gudjonsson JE . Genetic and immunological pathogenesis of atopic dermatitis. J Invest Dermatol. 2024;144(5):954‐968. 38085213 10.1016/j.jid.2023.10.019 PMC11040454

[103] Sroka‐Tomaszewska J , Trzeciak M . Molecular mechanisms of atopic dermatitis pathogenesis. Int J Mol Sci. 2021;22(8):4130. 33923629 10.3390/ijms22084130 PMC8074061

[104] Tsakok T , Woolf R , Smith CH , Weidinger S , Flohr C . Atopic dermatitis: the skin barrier and beyond. Br J Dermatol. 2019;180(3):464‐474. 29969827 10.1111/bjd.16934

[105] Agrawal R , Wisniewski JA , Woodfolk JA . The role of regulatory T cells in atopic dermatitis. Curr Probl Dermatol. 2011;41:112‐124. 21576952 10.1159/000323305 PMC4547455

[106] Kolukisa B , Baser D , Akcam B , et al. Evolution and long‐term outcomes of combined immunodeficiency due to CARMIL2 deficiency. Allergy. 2022;77(3):1004‐1019. 34287962 10.1111/all.15010 PMC9976932

[107] Malhotra N , Leyva‐Castillo JM , Jadhav U , et al. RORalpha‐expressing T regulatory cells restrain allergic skin inflammation. Sci Immunol. 2018;3(21):eaao6923. 29500225 10.1126/sciimmunol.aao6923 PMC5912895

[108] Papaioannou E , Yanez DC , Ross S , et al. Sonic hedgehog signaling limits atopic dermatitis via Gli2‐driven immune regulation. J Clin Invest. 2019;129(8):3153‐3170. 31264977 10.1172/JCI125170 PMC6668675

[109] Wilkie H , Janssen E , Leyva‐Castillo JM , Geha RS . DOCK8 expression in regulatory T cells maintains their stability and limits contact hypersensitivity. J Invest Dermatol. 2021;141(6):1503‐1511. 33171169 10.1016/j.jid.2020.09.027 PMC8102650

[110] Wilkie H , Das M , Pelovitz T , et al. Regulatory T‐cell dysfunction and cutaneous exposure to Staphylococcus aureus underlie eczema in DOCK8 deficiency. J Allergy Clin Immunol. 2024;154:143‐156. 38185418 10.1016/j.jaci.2023.12.020

[111] Jin S , Shin JU , Noh JY , et al. DOCK8: regulator of Treg in response to corticotropin‐releasing hormone. Allergy. 2016;71(6):811‐819. 26799599 10.1111/all.12845

[112] Nedoszytko B , Lange M , Sokolowska‐Wojdylo M , et al. The role of regulatory T cells and genes involved in their differentiation in pathogenesis of selected inflammatory and neoplastic skin diseases. Part II: the Treg role in skin diseases pathogenesis. Postepy Dermatol Alergol. 2017;34(5):405‐417. 29507554 10.5114/ada.2017.71105 PMC5835974

[113] Ito Y , Adachi Y , Makino T , et al. Expansion of FOXP3‐positive CD4+CD25+ T cells associated with disease activity in atopic dermatitis. Ann Allergy Asthma Immunol. 2009;103(2):160‐165. 19739430 10.1016/S1081-1206(10)60170-6

[114] Gaspar K , Barath S , Nagy G , et al. Regulatory T‐cell subsets with acquired functional impairment: important indicators of disease severity in atopic dermatitis. Acta Derm Venereol. 2015;95(2):151‐155. 24890798 10.2340/00015555-1882

[115] Zhang YY , Wang AX , Xu L , Shen N , Zhu J , Tu CX . Characteristics of peripheral blood CD4+CD25+ regulatory T cells and related cytokines in severe atopic dermatitis. Eur J Dermatol. 2016;26(3):240‐246. 27184163 10.1684/ejd.2015.2709

[116] Roesner LM , Floess S , Witte T , Olek S , Huehn J , Werfel T . Foxp3(+) regulatory T cells are expanded in severe atopic dermatitis patients. Allergy. 2015;70(12):1656‐1660. 26228301 10.1111/all.12712

[117] Looman KIM , van Meel ER , Grosserichter‐Wagener C , et al. Associations of Th2, Th17, Treg cells, and IgA(+) memory B cells with atopic disease in children: the generation R study. Allergy. 2020;75(1):178‐187. 31385614 10.1111/all.14010

[118] Stelmaszczyk‐Emmel A , Zawadzka‐Krajewska A , Szypowska A , Kulus M , Demkow U . Frequency and activation of CD4+CD25 FoxP3+ regulatory T cells in peripheral blood from children with atopic allergy. Int Arch Allergy Immunol. 2013;162(1):16‐24. 23817221 10.1159/000350769

[119] Zhang DJ , Hao F , Qian T , Cheng HX . Expression of helper and regulatory T cells in atopic dermatitis: a meta‐analysis. Front Pediatr. 2022;10:777992. 35433533 10.3389/fped.2022.777992 PMC9010508

[120] Mohr A , Malhotra R , Mayer G , Gorochov G , Miyara M . Human FOXP3(+) T regulatory cell heterogeneity. Clin Transl Immunology. 2018;7(1):e1005. 29484183 10.1002/cti2.1005 PMC5822410

[121] Passerini L , Allan SE , Battaglia M , et al. STAT5‐signaling cytokines regulate the expression of FOXP3 in CD4+CD25+ regulatory T cells and CD4+CD25‐ effector T cells. Int Immunol. 2008;20(3):421‐431. 18270368 10.1093/intimm/dxn002

[122] Ono M . Control of regulatory T‐cell differentiation and function by T‐cell receptor signalling and Foxp3 transcription factor complexes. Immunology. 2020;160(1):24‐37. 32022254 10.1111/imm.13178 PMC7160660

[123] Clark RA . Skin‐resident T cells: the ups and downs of on site immunity. J Invest Dermatol. 2010;130(2):362‐370. 19675575 10.1038/jid.2009.247 PMC2922675

[124] Vestergaard C , Bang K , Gesser B , Yoneyama H , Matsushima K , Larsen CG . A Th2 chemokine, TARC, produced by keratinocytes may recruit CLA+CCR4+ lymphocytes into lesional atopic dermatitis skin. J Invest Dermatol. 2000;115(4):640‐646. 10998136 10.1046/j.1523-1747.2000.00115.x

[125] Kakinuma T , Nakamura K , Wakugawa M , et al. Thymus and activation‐regulated chemokine in atopic dermatitis: serum thymus and activation‐regulated chemokine level is closely related with disease activity. J Allergy Clin Immunol. 2001;107(3):535‐541. 11240957 10.1067/mai.2001.113237

[126] Kim HJ , Kim YJ , Lee SH , Yu J , Jeong SK , Hong SJ . Effects of lactobacillus rhamnosus on allergic march model by suppressing Th2, Th17, and TSLP responses via CD4(+)CD25(+)Foxp3(+) Tregs. Clin Immunol. 2014;153(1):178‐186. 24769377 10.1016/j.clim.2014.04.008

[127] Reefer AJ , Satinover SM , Solga MD , et al. Analysis of CD25hiCD4+ “regulatory” T‐cell subtypes in atopic dermatitis reveals a novel T(H)2‐like population. J Allergy Clin Immunol. 2008;121(2):415‐422. 18177697 10.1016/j.jaci.2007.11.003

[128] Reefer AJ , Satinover SM , Wilson BB , Woodfolk JA . The relevance of microbial allergens to the IgE antibody repertoire in atopic and nonatopic eczema. J Allergy Clin Immunol. 2007;120(1):156‐163. 17507082 10.1016/j.jaci.2007.03.042

[129] Cheru N , Hafler DA , Sumida TS . Regulatory T cells in peripheral tissue tolerance and diseases. Front Immunol. 2023;14:1154575. 37197653 10.3389/fimmu.2023.1154575 PMC10183596

[130] Zhang Z , Guo J , Jia R . Treg plasticity and human diseases. Inflamm Res. 2023;72(12):2181‐2197. 37878023 10.1007/s00011-023-01808-x

[131] Kannan AK , Su Z , Gauvin DM , et al. IL‐23 induces regulatory T cell plasticity with implications for inflammatory skin diseases. Sci Rep. 2019;9(1):17675. 31776355 10.1038/s41598-019-53240-z PMC6881359

[132] Brandt EB , Sivaprasad U . Th2 cytokines and atopic dermatitis. J Clin Cell Immunol. 2011;2(3):110. 21994899 10.4172/2155-9899.1000110 PMC3189506

[133] Xu SX , McCormick JK . Staphylococcal superantigens in colonization and disease. Front Cell Infect Microbiol. 2012;2:52. 22919643 10.3389/fcimb.2012.00052 PMC3417409

[134] Cardona ID , Goleva E , Ou LS , Leung DY . Staphylococcal enterotoxin B inhibits regulatory T cells by inducing glucocorticoid‐induced TNF receptor‐related protein ligand on monocytes. J Allergy Clin Immunol. 2006;117(3):688‐695. 16522472 10.1016/j.jaci.2005.11.037

[135] Raeber ME , Sahin D , Karakus U , Boyman O . A systematic review of interleukin‐2‐based immunotherapies in clinical trials for cancer and autoimmune diseases. EBioMedicine. 2023;90:104539. 37004361 10.1016/j.ebiom.2023.104539 PMC10111960

[136] Satitsuksanoa P , Angelina A , Palomares O , Akdis M . Mechanisms in AIT: insights 2021. Allergol Select. 2022;6:259‐266. 36457721 10.5414/ALX02300E PMC9707368

[137] Berni Canani R , Caminati M , Carucci L , Eguiluz‐Gracia I . Skin, gut, and lung barrier: physiological interface and target of intervention for preventing and treating allergic diseases. Allergy. 2024;79:1485‐1500. 38439599 10.1111/all.16092

[138] Schuler CF , Lukacs NW , Baker JR Jr . Recent patents in allergy and immunology: Transepidermal water loss for anaphylaxis monitoring. Allergy. 2024;79(3):765‐766. 38205722 10.1111/all.16017 PMC10922900

[139] Stevens WW , Kraft M , Eisenbarth SC . Recent insights into the mechanisms of anaphylaxis. Curr Opin Immunol. 2023;81:102288. 36848746 10.1016/j.coi.2023.102288 PMC10023498

[140] Peng Z , Apfelbacher C , Brandstetter S , et al. Directed acyclic graph for epidemiological studies in childhood food allergy: construction, user's guide, and application. Allergy. 2024;79(8):2051‐2064. 38234010 10.1111/all.16025

[141] Riggioni C , Ricci C , Moya B , et al. Systematic review and meta‐analyses on the accuracy of diagnostic tests for IgE‐mediated food allergy. Allergy. 2024;79(2):324‐352. 38009299 10.1111/all.15939

[142] Santos AF , Riggioni C , Agache I , et al. EAACI guidelines on the diagnosis of IgE‐mediated food allergy. Allergy. 2023;78(12):3057‐3076. 37815205 10.1111/all.15902

[143] Tedner SG , Soderhall C , Konradsen JR , et al. Extract and molecular‐based early infant sensitization and associated factors‐a PreventADALL study. Allergy. 2021;76(9):2730‐2739. 33751598 10.1111/all.14805

[144] Pabst O , Mowat AM . Oral tolerance to food protein. Mucosal Immunol. 2012;5(3):232‐239. 22318493 10.1038/mi.2012.4 PMC3328017

[145] Kazmi W , Berin MC . Oral tolerance and oral immunotherapy for food allergy: evidence for common mechanisms? Cell Immunol. 2023;383:104650. 36543052 10.1016/j.cellimm.2022.104650

[146] Chen Y , Inobe J , Marks R , Gonnella P , Kuchroo VK , Weiner HL . Peripheral deletion of antigen‐reactive T cells in oral tolerance. Nature. 1995;376(6536):177‐180. 7603570 10.1038/376177a0

[147] Battais F , Mothes T , Moneret‐Vautrin DA , et al. Identification of IgE‐binding epitopes on gliadins for patients with food allergy to wheat. Allergy. 2005;60(6):815‐821. 15876313 10.1111/j.1398-9995.2005.00795.x

[148] Hong SW , Krueger PD , Osum KC , et al. Immune tolerance of food is mediated by layers of CD4(+) T cell dysfunction. Nature. 2022;607(7920):762‐768. 35794484 10.1038/s41586-022-04916-6 PMC10336534

[149] Lozano‐Ojalvo D , Tyler SR , Aranda CJ , et al. Allergen recognition by specific effector Th2 cells enables IL‐2‐dependent activation of regulatory T‐cell responses in humans. Allergy. 2023;78(3):697‐713. 36089900 10.1111/all.15512 PMC10111618

[150] Bonnet B , Vigneron J , Levacher B , et al. Low‐dose IL‐2 induces regulatory T cell‐mediated control of experimental food allergy. J Immunol. 2016;197(1):188‐198. 27259854 10.4049/jimmunol.1501271

[151] Klein M , Misme‐Aucouturier B , Cheminant MA , et al. Engineering a safe monoclonal anti‐human IL‐2 that is effective in a murine model of food allergy and asthma. Allergy. 2022;77(3):933‐945. 34324715 10.1111/all.15029

[152] Hadis U , Wahl B , Schulz O , et al. Intestinal tolerance requires gut homing and expansion of FoxP3+ regulatory T cells in the lamina propria. Immunity. 2011;34(2):237‐246. 21333554 10.1016/j.immuni.2011.01.016

[153] Mucida D , Kutchukhidze N , Erazo A , Russo M , Lafaille JJ , Curotto de Lafaille MA . Oral tolerance in the absence of naturally occurring Tregs. J Clin Invest. 2005;115(7):1923‐1933. 15937545 10.1172/JCI24487 PMC1142115

[154] Worbs T , Bode U , Yan S , et al. Oral tolerance originates in the intestinal immune system and relies on antigen carriage by dendritic cells. J Exp Med. 2006;203(3):519‐527. 16533884 10.1084/jem.20052016 PMC2118247

[155] Mazzini E , Massimiliano L , Penna G , Rescigno M . Oral tolerance can be established via gap junction transfer of fed antigens from CX3CR1(+) macrophages to CD103(+) dendritic cells. Immunity. 2014;40(2):248‐261. 24462723 10.1016/j.immuni.2013.12.012

[156] Rivera CA , Randrian V , Richer W , et al. Epithelial colonization by gut dendritic cells promotes their functional diversification. Immunity. 2022;55(1):129‐144. 34910930 10.1016/j.immuni.2021.11.008 PMC8751639

[157] Dawicki W , Li C , Town J , Zhang X , Gordon JR . Therapeutic reversal of food allergen sensitivity by mature retinoic acid‐differentiated dendritic cell induction of LAG3(+)CD49b(−)Foxp3(−) regulatory T cells. J Allergy Clin Immunol. 2017;139(5):1608‐1620. 28277274 10.1016/j.jaci.2016.07.042

[158] Wu R , Yuan X , Li X , et al. The bile acid‐activated retinoic acid response in dendritic cells is involved in food allergen sensitization. Allergy. 2022;77(2):483‐498. 34365653 10.1111/all.15039

[159] Iwata M , Hirakiyama A , Eshima Y , Kagechika H , Kato C , Song SY . Retinoic acid imprints gut‐homing specificity on T cells. Immunity. 2004;21(4):527‐538. 15485630 10.1016/j.immuni.2004.08.011

[160] Kutyavin V , Korn LL , Medzhitov R . Nutrient‐derived signals regulate eosinophil adaptation to the small intestine. Proc Natl Acad Sci USA. 2024;121(5):e2316446121. 38271336 10.1073/pnas.2316446121 PMC10835075

[161] Shaikh N , Waterholter A , Gnirck AC , et al. Retinoic acid drives intestine‐specific adaptation of effector ILC2s originating from distant sites. J Exp Med. 2023;220(12):e20221015. 37773047 10.1084/jem.20221015 PMC10541314

[162] Olin A , Henckel E , Chen Y , et al. Stereotypic immune system development in newborn children. Cell. 2018;174(5):1277‐1292. 30142345 10.1016/j.cell.2018.06.045 PMC6108833

[163] Sanidad KZ , Rager SL , Carrow HC , et al. Gut bacteria‐derived serotonin promotes immune tolerance in early life. Sci Immunol. 2024;9(93):eadj4775. 38489352 10.1126/sciimmunol.adj4775 PMC11328322

[164] Al Nabhani Z , Dulauroy S , Marques R , et al. A weaning reaction to microbiota is required for resistance to Immunopathologies in the adult. Immunity. 2019;50(5):1276‐1288. 30902637 10.1016/j.immuni.2019.02.014

[165] Ramanan D , Pratama A , Zhu Y , et al. Regulatory T cells in the face of the intestinal microbiota. Nat Rev Immunol. 2023;23(11):749‐762. 37316560 10.1038/s41577-023-00890-w

[166] Akagbosu B , Tayyebi Z , Shibu G , et al. Novel antigen‐presenting cell imparts T(reg)‐dependent tolerance to gut microbiota. Nature. 2022;610(7933):752‐760. 36070798 10.1038/s41586-022-05309-5 PMC9605865

[167] Kedmi R , Najar TA , Mesa KR , et al. A RORgammat(+) cell instructs gut microbiota‐specific T(reg) cell differentiation. Nature. 2022;610(7933):737‐743. 36071167 10.1038/s41586-022-05089-y PMC9908423

[168] Lyu M , Suzuki H , Kang L , et al. ILC3s select microbiota‐specific regulatory T cells to establish tolerance in the gut. Nature. 2022;610(7933):744‐751. 36071169 10.1038/s41586-022-05141-x PMC9613541

[169] Verma R , Lee C , Jeun EJ , et al. Cell surface polysaccharides of Bifidobacterium bifidum induce the generation of Foxp3(+) regulatory T cells. Sci Immunol. 2018;3(28):eaat6975. 30341145 10.1126/sciimmunol.aat6975

[170] Song X , Sun X , Oh SF , et al. Microbial bile acid metabolites modulate gut RORgamma(+) regulatory T cell homeostasis. Nature. 2020;577(7790):410‐415. 31875848 10.1038/s41586-019-1865-0 PMC7274525

[171] Bunyavanich S , Berin MC . Food allergy and the microbiome: current understandings and future directions. J Allergy Clin Immunol. 2019;144(6):1468‐1477. 31812181 10.1016/j.jaci.2019.10.019 PMC6905201

[172] Rachid R , Stephen‐Victor E , Chatila TA . The microbial origins of food allergy. J Allergy Clin Immunol. 2021;147(3):808‐813. 33347905 10.1016/j.jaci.2020.12.624 PMC8096615

[173] Feehley T , Plunkett CH , Bao R , et al. Healthy infants harbor intestinal bacteria that protect against food allergy. Nat Med. 2019;25(3):448‐453. 30643289 10.1038/s41591-018-0324-z PMC6408964

[174] Noval Rivas M , Burton OT , Wise P , et al. Regulatory T cell reprogramming toward a Th2‐cell‐like lineage impairs oral tolerance and promotes food allergy. Immunity. 2015;42(3):512‐523. 25769611 10.1016/j.immuni.2015.02.004 PMC4366316

[175] Burton OT , Logsdon SL , Zhou JS , et al. Oral immunotherapy induces IgG antibodies that act through FcgammaRIIb to suppress IgE‐mediated hypersensitivity. J Allergy Clin Immunol. 2014;134(6):1310‐1317. 25042981 10.1016/j.jaci.2014.05.042 PMC4261076

[176] Gowthaman U , Chen JS , Zhang B , et al. Identification of a T follicular helper cell subset that drives anaphylactic IgE. Science. 2019;365(6456):eaaw6433. 31371561 10.1126/science.aaw6433 PMC6901029

[177] Gonzalez‐Figueroa P , Roco JA , Papa I , et al. Follicular regulatory T cells produce neuritin to regulate B cells. Cell. 2021;184(7):1775‐1789. 33711260 10.1016/j.cell.2021.02.027

[178] Clement RL , Daccache J , Mohammed MT , et al. Follicular regulatory T cells control humoral and allergic immunity by restraining early B cell responses. Nat Immunol. 2019;20(10):1360‐1371. 31477921 10.1038/s41590-019-0472-4 PMC6754271

[179] Levy ML , Bacharier LB , Bateman E , et al. Key recommendations for primary care from the 2022 global initiative for asthma (GINA) update. NPJ Prim Care Respir Med. 2023;33(1):7. 36754956 10.1038/s41533-023-00330-1 PMC9907191

[180] Wenzel SE . Asthma phenotypes: the evolution from clinical to molecular approaches. Nat Med. 2012;18(5):716‐725. 22561835 10.1038/nm.2678

[181] Sporik R , Platts‐Mills TA . Allergen exposure and the development of asthma. Thorax. 2001;56(Suppl 2):ii58‐ii63. 11514708 PMC1765986

[182] Komlosi ZI , van de Veen W , Kovacs N , et al. Cellular and molecular mechanisms of allergic asthma. Mol Asp Med. 2022;85:100995. 10.1016/j.mam.2021.100995 34364680

[183] Palomares O , Akdis M , Martin‐Fontecha M , Akdis CA . Mechanisms of immune regulation in allergic diseases: the role of regulatory T and B cells. Immunol Rev. 2017;278(1):219‐236. 28658547 10.1111/imr.12555

[184] Suto A , Nakajima H , Kagami SI , Suzuki K , Saito Y , Iwamoto I . Role of CD4(+) CD25(+) regulatory T cells in T helper 2 cell‐mediated allergic inflammation in the airways. Am J Respir Crit Care Med. 2001;164(4):680‐687. 11520737 10.1164/ajrccm.164.4.2010170

[185] Kraszula L , Eusebio MO , Kuna P , Pietruczuk M . Relationship between CCR5(+)FoxP3(+) Treg cells and forced expiratory volume in 1 s, peak expiratory flow in patients with severe asthma. Postepy Dermatol Alergol. 2021;38(2):262‐268. 34408594 10.5114/ada.2021.106202 PMC8362744

[186] Hartl D , Koller B , Mehlhorn AT , et al. Quantitative and functional impairment of pulmonary CD4+CD25hi regulatory T cells in pediatric asthma. J Allergy Clin Immunol. 2007;119(5):1258‐1266. 17412402 10.1016/j.jaci.2007.02.023

[187] Nguyen KD , Vanichsarn C , Fohner A , Nadeau KC . Selective deregulation in chemokine signaling pathways of CD4+CD25(hi)CD127(lo)/(−) regulatory T cells in human allergic asthma. J Allergy Clin Immunol. 2009;123(4):933‐939. 19152963 10.1016/j.jaci.2008.11.037 PMC4214553

[188] Chantveerawong T , Sangkangjanavanich S , Chiewchalermsri C , et al. Increased circulating CRTH2(+) Tregs are associated with asthma control and exacerbation. Allergy. 2022;77(2):681‐685. 34676900 10.1111/all.15145

[189] Ntontsi P , Photiades A , Zervas E , Xanthou G , Samitas K . Genetics and epigenetics in asthma. Int J Mol Sci. 2021;22(5):2412. 33673725 10.3390/ijms22052412 PMC7957649

[190] Schoettler N , Rodriguez E , Weidinger S , Ober C . Advances in asthma and allergic disease genetics: is bigger always better? J Allergy Clin Immunol. 2019;144(6):1495‐1506. 31677964 10.1016/j.jaci.2019.10.023 PMC6900451

[191] Demenais F , Margaritte‐Jeannin P , Barnes KC , et al. Multiancestry association study identifies new asthma risk loci that colocalize with immune‐cell enhancer marks. Nat Genet. 2018;50(1):42‐53. 29273806 10.1038/s41588-017-0014-7 PMC5901974

[192] Torgerson DG , Ampleford EJ , Chiu GY , et al. Meta‐analysis of genome‐wide association studies of asthma in ethnically diverse north American populations. Nat Genet. 2011;43(9):887‐892. 21804549 10.1038/ng.888 PMC3445408

[193] Olafsdottir TA , Theodors F , Bjarnadottir K , et al. Eighty‐eight variants highlight the role of T cell regulation and airway remodeling in asthma pathogenesis. Nat Commun. 2020;11(1):393. 31959851 10.1038/s41467-019-14144-8 PMC6971247

[194] Bennett CL , Christie J , Ramsdell F , et al. The immune dysregulation, polyendocrinopathy, enteropathy, X‐linked syndrome (IPEX) is caused by mutations of FOXP3. Nat Genet. 2001;27(1):20‐21. 11137993 10.1038/83713

[195] Ding L , Abebe T , Beyene J , et al. Rank‐based genome‐wide analysis reveals the association of ryanodine receptor‐2 gene variants with childhood asthma among human populations. Hum Genomics. 2013;7(1):16. 23829686 10.1186/1479-7364-7-16 PMC3708719

[196] Croisier H , Tan X , Chen J , Sneyd J , Sanderson MJ , Brook BS . Ryanodine receptor sensitization results in abnormal calcium signaling in airway smooth muscle cells. Am J Respir Cell Mol Biol. 2015;53(5):703‐711. 25874477 10.1165/rcmb.2014-0386OC PMC4742950

[197] Uchiyama Y , Murakami G , Ohno Y . The fine structure of nerve endings on rat thyroid follicular cells. Cell Tissue Res. 1985;242(2):457‐460. 4053175 10.1007/BF00214563

[198] Wang X , Geng S , Meng J , et al. Foxp3‐mediated blockage of ryanodine receptor 2 underlies contact‐based suppression by regulatory T cells. J Clin Invest. 2023;133(24):e163470. 38099494 10.1172/JCI163470 PMC10721146

[199] Gern JE . How rhinovirus infections cause exacerbations of asthma. Clin Exp Allergy. 2015;45(1):32‐42. 25270551 10.1111/cea.12428

[200] Jansen K , Wirz OF , van de Veen W , et al. Loss of regulatory capacity in Treg cells following rhinovirus infection. J Allergy Clin Immunol. 2021;148(4):1016‐1029. 34153372 10.1016/j.jaci.2021.05.045

[201] Nadeau K , McDonald‐Hyman C , Noth EM , et al. Ambient air pollution impairs regulatory T‐cell function in asthma. J Allergy Clin Immunol. 2010;126(4):845‐852. 20920773 10.1016/j.jaci.2010.08.008

[202] Prunicki M , Cauwenberghs N , Lee J , et al. Air pollution exposure is linked with methylation of immunoregulatory genes, altered immune cell profiles, and increased blood pressure in children. Sci Rep. 2021;11(1):4067. 33603036 10.1038/s41598-021-83577-3 PMC7893154

[203] Hew KM , Walker AI , Kohli A , et al. Childhood exposure to ambient polycyclic aromatic hydrocarbons is linked to epigenetic modifications and impaired systemic immunity in T cells. Clin Exp Allergy. 2015;45(1):238‐248. 25048800 10.1111/cea.12377 PMC4396982

[204] Sun L , Fu J , Lin SH , et al. Particulate matter of 2.5 mum or less in diameter disturbs the balance of T(H)17/regulatory T cells by targeting glutamate oxaloacetate transaminase 1 and hypoxia‐inducible factor 1alpha in an asthma model. J Allergy Clin Immunol. 2020;145(1):402‐414. 31647966 10.1016/j.jaci.2019.10.008

[205] Xia M , Viera‐Hutchins L , Garcia‐Lloret M , et al. Vehicular exhaust particles promote allergic airway inflammation through an aryl hydrocarbon receptor‐notch signaling cascade. J Allergy Clin Immunol. 2015;136(2):441‐453. 25825216 10.1016/j.jaci.2015.02.014 PMC4530027

[206] Benamar M , Harb H , Chen Q , et al. A common IL‐4 receptor variant promotes asthma severity via a T(reg) cell GRB2‐IL‐6‐Notch4 circuit. Allergy. 2022;77(11):3377‐3387. 35841382 10.1111/all.15444 PMC9617759

[207] Doganci A , Eigenbrod T , Krug N , et al. The IL‐6R alpha chain controls lung CD4+CD25+ Treg development and function during allergic airway inflammation in vivo. J Clin Invest. 2005;115(2):313‐325. 15668741 10.1172/JCI22433 PMC544603

[208] Esty B , Harb H , Bartnikas LM , et al. Treatment of severe persistent asthma with IL‐6 receptor blockade. J Allergy Clin Immunol Pract. 2019;7(5):1639‐1642. 30885880 10.1016/j.jaip.2019.02.043 PMC6511285

[209] MacBeth M , Joetham A , Gelfand EW , Schedel M . Plasticity of naturally occurring regulatory T cells in allergic airway disease is modulated by the transcriptional activity of Il‐6. Int J Mol Sci. 2021;22(9):4582. 33925531 10.3390/ijms22094582 PMC8123826

[210] Kahhaleh FG , Barrientos G , Conrad ML . The gut‐lung axis and asthma susceptibility in early life. Acta Physiol (Oxf). 2024;240(3):e14092. 38251788 10.1111/apha.14092

[211] Johnson JL , Jones MB , Cobb BA . Polysaccharide‐experienced effector T cells induce IL‐10 in FoxP3+ regulatory T cells to prevent pulmonary inflammation. Glycobiology. 2018;28(1):50‐58. 29087497 10.1093/glycob/cwx093 PMC5972631

[212] Arpaia N , Campbell C , Fan X , et al. Metabolites produced by commensal bacteria promote peripheral regulatory T‐cell generation. Nature. 2013;504(7480):451‐455. 24226773 10.1038/nature12726 PMC3869884

[213] Smith PM , Howitt MR , Panikov N , et al. The microbial metabolites, short‐chain fatty acids, regulate colonic Treg cell homeostasis. Science. 2013;341(6145):569‐573. 23828891 10.1126/science.1241165 PMC3807819

[214] Furusawa Y , Obata Y , Fukuda S , et al. Commensal microbe‐derived butyrate induces the differentiation of colonic regulatory T cells. Nature. 2013;504(7480):446‐450. 24226770 10.1038/nature12721

[215] Park J , Kim M , Kang SG , et al. Short‐chain fatty acids induce both effector and regulatory T cells by suppression of histone deacetylases and regulation of the mTOR‐S6K pathway. Mucosal Immunol. 2015;8(1):80‐93. 24917457 10.1038/mi.2014.44 PMC4263689

[216] Johnson JL , Jones MB , Cobb BA . Polysaccharide a from the capsule of Bacteroides fragilis induces clonal CD4+ T cell expansion. J Biol Chem. 2015;290(8):5007‐5014. 25540199 10.1074/jbc.M114.621771 PMC4335237

[217] Hooi JKY , Lai WY , Ng WK , et al. Global prevalence of helicobacter pylori infection: systematic review and meta‐analysis. Gastroenterology. 2017;153(2):420‐429. 28456631 10.1053/j.gastro.2017.04.022

[218] Chen Y , Blaser MJ . Inverse associations of helicobacter pylori with asthma and allergy. Arch Intern Med. 2007;167(8):821‐827. 17452546 10.1001/archinte.167.8.821

[219] Chen Y , Blaser MJ . Helicobacter pylori colonization is inversely associated with childhood asthma. J Infect Dis. 2008;198(4):553‐560. 18598192 10.1086/590158 PMC3902975

[220] Melby KK , Carlsen KL , Haland G , Samdal HH , Carlsen KH . Helicobacter pylori in early childhood and asthma in adolescence. BMC Res Notes. 2020;13(1):79. 32070394 10.1186/s13104-020-04941-6 PMC7027323

[221] Lundgren A , Stromberg E , Sjoling A , et al. Mucosal FOXP3‐expressing CD4+ CD25high regulatory T cells in helicobacter pylori‐infected patients. Infect Immun. 2005;73(1):523‐531. 15618192 10.1128/IAI.73.1.523-531.2005 PMC538965

[222] Owyang SY , Zhang M , El‐Zaatari M , et al. Dendritic cell‐derived TGF‐beta mediates the induction of mucosal regulatory T‐cell response to helicobacter infection essential for maintenance of immune tolerance in mice. Helicobacter. 2020;25(6):e12763. 33025641 10.1111/hel.12763 PMC7885176

[223] Oertli M , Noben M , Engler DB , et al. Helicobacter pylori gamma‐glutamyl transpeptidase and vacuolating cytotoxin promote gastric persistence and immune tolerance. Proc Natl Acad Sci USA. 2013;110(8):3047‐3052. 23382221 10.1073/pnas.1211248110 PMC3581963

[224] Arnold IC , Dehzad N , Reuter S , et al. Helicobacter pylori infection prevents allergic asthma in mouse models through the induction of regulatory T cells. J Clin Invest. 2011;121(8):3088‐3093. 21737881 10.1172/JCI45041 PMC3148731

[225] Oertli M , Sundquist M , Hitzler I , et al. DC‐derived IL‐18 drives Treg differentiation, murine helicobacter pylori‐specific immune tolerance, and asthma protection. J Clin Invest. 2012;122(3):1082‐1096. 22307326 10.1172/JCI61029 PMC3287234

[226] Engler DB , Reuter S , van Wijck Y , et al. Effective treatment of allergic airway inflammation with helicobacter pylori immunomodulators requires BATF3‐dependent dendritic cells and IL‐10. Proc Natl Acad Sci USA. 2014;111(32):11810‐11815. 25074917 10.1073/pnas.1410579111 PMC4136625

[227] Kyburz A , Fallegger A , Zhang X , et al. Transmaternal helicobacter pylori exposure reduces allergic airway inflammation in offspring through regulatory T cells. J Allergy Clin Immunol. 2019;143(4):1496‐1512. 30240703 10.1016/j.jaci.2018.07.046 PMC6592617

[228] Reuter S , Raspe J , Uebner H , et al. Treatment with helicobacter pylori‐derived VacA attenuates allergic airway disease. Front Immunol. 2023;14:1092801. 36761723 10.3389/fimmu.2023.1092801 PMC9902502

[229] Raspe J , Schmitz MS , Barbet K , et al. Therapeutic properties of helicobacter pylori‐derived vacuolating cytotoxin a in an animal model of chronic allergic airway disease. Respir Res. 2023;24(1):178. 37415170 10.1186/s12931-023-02484-5 PMC10324189

